# Echinocandin Adaptation in *Candida albicans* Is Accompanied by Altered Chromatin Accessibility at Gene Promoters and by Cell Wall Remodeling

**DOI:** 10.3390/jof11020110

**Published:** 2025-02-01

**Authors:** Sudisht K. Sah, Anshuman Yadav, Tyler Stahl, Jeffrey J. Hayes, Michael Bulger, Elena Rustchenko

**Affiliations:** 1 Department of Biochemistry and Biophysics, University of Rochester Medical Center, Rochester, NY 14642, USA; sudisht_sah@urmc.rochester.edu (S.K.S.); anshumanbiochem@gmail.com (A.Y.); jeffrey_hayes@urmc.rochester.edu (J.J.H.); 2 Genomic Research Center, University of Rochester Medical Center, Rochester, NY 14642, USA; tyler_stahl@urmc.rochester.edu; 3 Center for Pediatric Biochemical Research, Department of Pediatrics, University of Rochester Medical Center, Rochester, NY 14642, USA; michael_bulger@urmc.rochester.edu

**Keywords:** *Candida albicans*, ATAC-seq, echinocandin adaptation

## Abstract

Infections by the major opportunistic pathogen of human *Candida albicans* are commonly treated with echinocandin (ECN) drugs. However, *C. albicans* can adapt to grow in the presence of certain amounts of ECNs. Prior studies by several laboratories have defined multiple genes, as well as mechanisms involving induced aneuploidy, that can govern this. Still, the mechanisms of ECN adaptation are not fully understood. Here, we use genome-wide profiling of chromatin accessibility by ATAC-seq to determine if ECN adaptation is reflected in changes in the chromatin landscape in the absence of aneuploidy. We find that drug adaptation is coupled with multiple changes in chromatin accessibility genome-wide, which occur predominantly in gene promoter regions. Areas of increased accessibilities in promoters are enriched with the binding motifs for at least two types of transcription factors: zinc finger and basic leucine zipper. We also find that chromatin changes are often associated with differentially expressed genes including genes with functions relevant to the ECN-adapted phenotype, such as cell wall biosynthesis. Consistent with this, we find that the cell wall is remodeled in ECN-adapted mutants, with chitin up and glucan down and increased cell surface exposure. A full understanding of ECN adaptation processes is of critical importance for the prevention of clinical resistance.

## 1. Introduction

The occurrence of invasive fungal diseases is increasing worldwide, particularly among immunocompromised individuals (reviewed in [1,2,3]). *Candida* species responsible for invasive candidiasis are a major contributor to overall human fungal infections [4,5]. The opportunistic human pathogen *Candida albicans* is the leading cause of systemic fungal infections in North America and is included in the list of Fungal Priority Pathogens by the World Health Organization (reviewed in [6,7]).

The front-line echinocandin (ECN) drugs caspofungin, anidulafungin, micafungin, and rezafungin act by interfering with the biosynthesis of the cell wall of *C*. *albicans* via inhibition of 1,3-*β*-D-glucan synthase [8,9]. As the use of ECN drugs has increased in the last decade, the occurrence of clinical resistance in *C. albicans* has also increased [5,8,10,11,12]. Clinical resistance of *C. albicans* to ECNs is generally due to point mutations in the *FKS1* (orf19.2929) gene residing on chromosome 1 (Ch1) that codes for a catalytic subunit of 1,3-*β*-D-glucan synthase [13]. However, multiple *C. albicans* clinical isolates that lack *FKS1* resistance mutations nevertheless display a wide range of ECN minimum inhibitory concentrations (MICs), up to the clinical breakpoint (reviewed in [14,15]). This physiological state, which can be characterized with in vitro assays by increased ECN MICs in the absence of *FKS1* mutations, we hereafter refer to as adaptation.

Mechanisms involved in decreasing the susceptibility of *C. albicans* to ECNs are of considerable importance, and are likely to provide critical steps in the evolution of clinical resistance [16]. Prior studies from different laboratories have identified multiple genes involved in ECN susceptibility, with some shown to be relevant in clinical isolates [17]; (reviewed in [14,18]). In addition, aneuploidy of *C. albicans* Ch2 or Ch5 was shown to represent some mechanisms of adaptation (reviewed in [18]). Direct evidence of small decreases in susceptibility preceding clinical resistance due to *FKS* mutation was provided using a related *C. glabrata* [19,20]. Still, the full range of molecular mechanisms involved in adaptation, leading up to clinical ECN resistance, remains to be defined.

We have previously generated caspofungin-adapted mutants that model clinical isolates exhibiting elevated MICs in the absence of *FKS1* mutations [21]. Aneuploidy—loss of one Ch5 or the formation of iso-chromosome of Ch5 having two right arms—was observed in many of these mutants. Most isolated mutants, however, adapted via an aneuploidy-independent mechanism. In the mutants from all of the above classes, we identified point mutations including mutational hot spots, sometimes shared, as well as other changes to genomic DNA, along with multiple differentially expressed genes (DEGs) [14,22], suggesting that the mechanisms of adaptation could be complex. Notably, in fission yeast, increased resistance to drugs is often associated with changes in gene expression that are accomplished via epigenetic, chromatin-mediated mechanisms [23].

To address the possible role of chromatin structure in mechanisms contributing to adaptation to ECNs in *C. albicans*, we employed the Assay for Transposase-Accessible Chromatin with high-throughput sequencing (ATAC-seq) and determined the relationship between changes in chromatin accessibility and adaptation to ECN drugs in aneuploidy-independent adapted mutants.

## 2. Results

Genome-wide changes in chromatin accessibility in ECN-adapted mutants. We asked whether changes in chromatin accessibility occurred in *C. albicans* cells that have adapted to ECN drugs in the absence of aneuploidy. We performed ATAC-seq analysis with two euploid-adapted mutants, JMC200-2-5 and JMC160-2-5, which were derived from the parental strain JRCT1 by independent mutational events on solid medium supplemented with caspofungin [21]. These mutants became adapted to all three ECNs tested (caspofungin, anidulafungin, and micafungin) [24]; Section 4.

**Figure 1:**
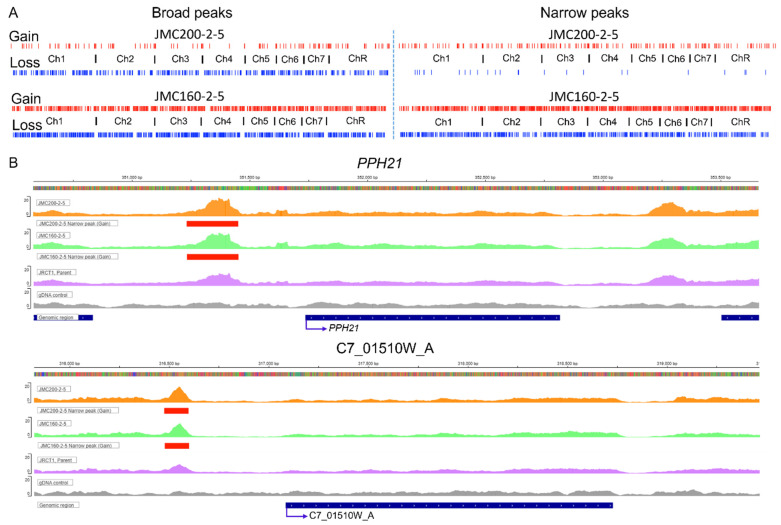
Characteristics of significant ATAC-seq peaks in ECN-adapted mutants JMC200-2-5 and JMC160-2-5 vs. parental strain JRCT1: (**A**). Distribution of ATAC-seq peaks throughout chromosomes. Red and blue tracks represent gain or loss of peaks as indicated. (**B**). Snapshot showing an example of IGV-track with gain of narrow peak in the promoter area of *PPH21* and C7_01510W_A (red) in both adapted mutants. Presented are mutants JMC160-2-5 and JMC200-2-5, parental JRCT1, and control gDNA, as indicated. See Figure S1 for examples of the gain of broad peaks, as well as a loss of narrow and broad peaks in *PGA25*, *PGA63*, *LSP1*, *ECM21*, *PHO87*, *CAN2*, *GLC3*, *BMT7*, *MKC1*, *FUN31*, *CRZ1*, *RIM101*, *CUP9*, and *CHS1*.

By using MACS2, DiffBind, and ChIPseeker packages (Section 4), we found a large number of areas of increased or decreased read coverage genome-wide in both mutants. Such areas indicate an increase or decrease in chromatin accessibility and are usually associated with gained or lost peaks [25,26]. According to the MACS2 peak threshold applied, the JMC200-2-5 strain exhibits 890 differential peaks, while JMC160-2-5 exhibits 4121, as compared to the parental strain (Figure 1A, Table 1; Tables S1–S4). In JMC200-2-5417 peaks are gained, while 473 are lost, while in JMC160-2-5, substantially more peaks are gained, 2464, than lost, 1657. In both strains, differential ATAC-seq peaks are distributed in a non-biased manner across all eight *C. albicans* chromosomes (Figure 1A, Table 1).

**Figure 2:**
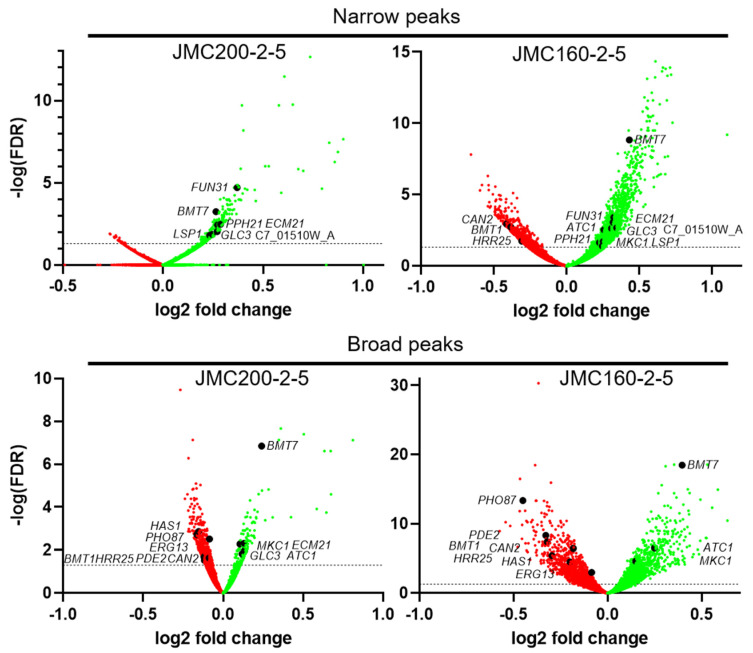
Volcano plots of Narrow and Broad ATAC-seq peaks in ECN-adapted mutants JMC200-2-5 and JMC160-2-5 vs. parental strain JRCT1, as indicated. The y-axis represents the negative log10 adjusted *p*-values (false discovery rate, FDR). The x-axis represents the log2 fold change between parent and mutant. The horizontal dashed line indicates FDR equal to 0.05. Each dot represents a peak annotated to the nearest gene. Red stands for lost peaks, while green stands for gained peaks. Black dots indicate 16 caspofungin responsive and cell wall build genes. Images were generated using GraphPad Prism version 9.5.0 for Windows.

Peaks called from ATAC-seq datasets can be classified as either Narrow or Broad, as determined by MACS2 (Section 4). Notably, ATAC-seq peaks associated with transcription factor (TF) binding generally appear as narrow peaks, while peaks associated with posttranslational histone modifications exhibit broader peaks [27]. Interestingly, JMC160-2-5 loses a large number of narrow peaks (734), unlike JMC200-2-5, which loses only 26 narrow peaks (Table 1). Notably, however, these changes were quantitative, and not reflected in complete gain or loss of individual peaks. Examples of peaks that exhibited quantitative changes in intensity sufficient to be called “gained” or “lost” are shown in Figure 1B, while Figure 2 summarizes the differential peaks, including Broad and Narrow categories in both mutants.

**Figure 3:**
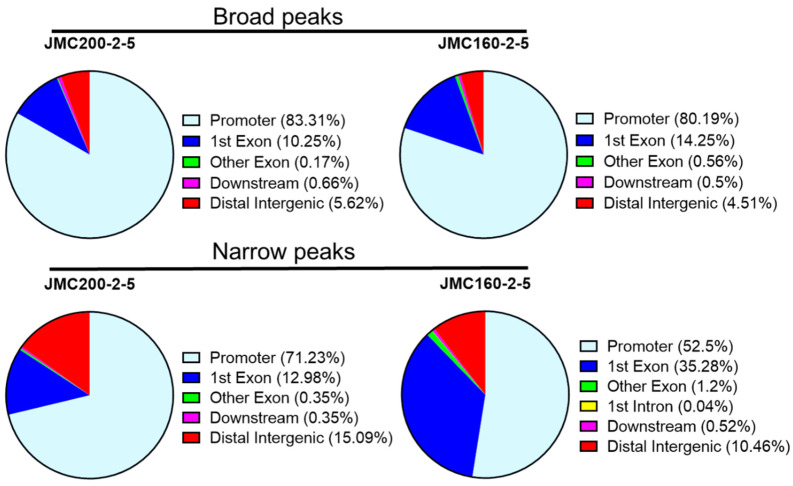
Pie chart showing the distribution of significant ATAC-seq peaks over different genomic regions according to program ChIPSeeker (see Section 4).

To address the distribution of significant ATAC-seq peaks genome-wide, we used the ChIPseeker Bioconductor package “annotatePeaks” function, an approach that was previously applied to a study of *C. albicans* under oxidative stress ([28]; Section 4). This method identifies significant changes in the chromatin landscape of such regions as Promoter, 1st Exon, Other Exon, Downstream (≤300 bp), or Distal intergenic. We found that ATAC-seq peaks are distributed non-randomly, with most located at gene promoter regions (Figure 3).

Differential ATAC-seq peaks are associated with differentially expressed genes (DEGs). Our initial ATAC-seq analysis indicates that the two ECN-adapted *C. albicans* mutant strains exhibit changes in chromatin accessibility genome-wide as compared to the parental strain. We next compared these changes to changes in gene expression, using our previously published transcriptional profiles of JMC200-2-5 and JMC160-2-5 (Section 4; Tables S5 and S6) [14]. We find a general correlation between the number of differential ATAC-seq peaks and DEGs. JMC160-2-5 harbors a larger number of DEGs (3135 out of 6182 genes, ~51% of total genes) and a larger number of differential ATAC-seq peaks (4121), while JMC200-2-5 harbors a smaller number of DEGs (2664, ~43%) and differential peaks (890) (Table 2).

To further probe the relationship between differential ATAC-seq peaks and DEGs, we used the ChIPseeker Bioconductor package “annotatePeaks” function (tssRegion = c(−1000, 200), genomicAnnotationPriority = c(‘Promoter’, ‘5UTR’, ‘3UTR’, ‘Exon’, ‘Intron’, ‘Downstream’, ‘Intergenic’) (Section 4) that annotates peaks to the nearest gene based on proximity to the transcriptional start site (TSS). We then looked for associations between differential ATAC-seq peaks and DEGs that changed in the same direction (both filtered for *p*-adj < 0.05). To identify overlapping genes associated with both differential ATAC-seq peaks and DEGs, we utilized Python to find the intersection between the two sets of genes. We find that 216 of 890 differential ATAC-seq peaks (24.26%) in the JMC160-2-5 mutant and 965 of 4121 peaks (23.41%) in the JMC200-2-5 mutant occur in proximity to and change in the same direction as DEGs (Table 2).

**Figure 4:**
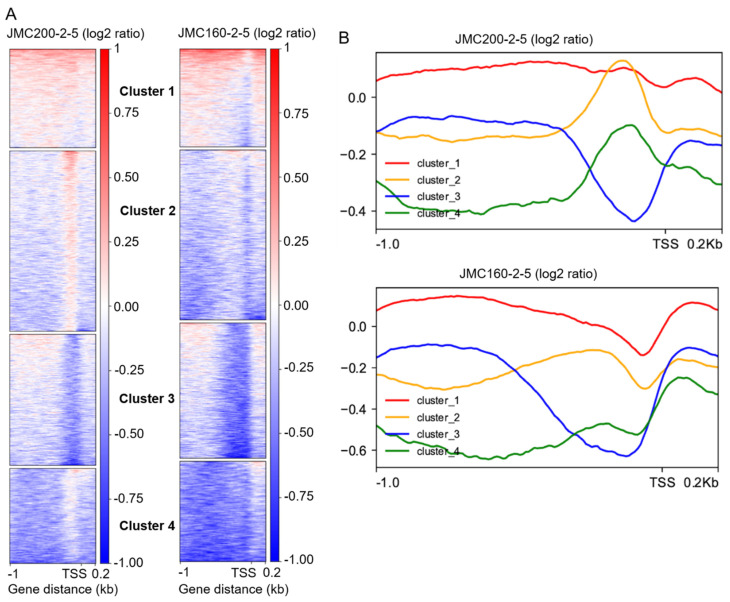
Characteristics of ATAC-seq peaks that are found in all promoter regions of ECN-adapted mutants JMC200-2-5 and JMC160-2-5 vs. parental strain JRCT1: (**A**). Heat maps of all peaks according to program deepTools (see Section 4) within the range of −1000/+200 bp relative to TSS. Shown are gain (red) and loss (blue) of peaks that are clustered according to K-means clustering of differential ATAC-seq nucleosome-free read coverage (log2-fold change). Note that ATAC-seq reads are split into clusters 1, 2, 3, and 4 of which the top cluster is enriched with gain of peaks. (**B**). Average plot of each cluster of both mutants.

Analysis of accessibility changes at or near transcriptional start sites (TSSs). In order to better assess accessibility changes at or near promoter regions, we continued analyzing our data with an approach used for *C. albicans* previously [28]. We performed K-means clustering of all promoter regions (−1000/+200 bp around the TSS) based on their differential read coverage profile compared to the parental strain. This analysis generates four clusters in both mutants (Figure 4). For both mutants, Cluster 1 consists of promoters that exhibit a generalized increase in accessibility across the entire region, while Cluster 2 consists of promoters that exhibit a generalized, but moderate decrease in accessibility. In the JMC200-2-5 strain, promoters grouped into Cluster 2 also exhibit a localized increase in accessibility at approximately −200 bp. Cluster 3 is defined by a dramatic decrease in chromatin accessibility localized to the region of 200 bp upstream of TSS, while Cluster 4 consists of promoters exhibiting a dramatic decrease in chromatin accessibility throughout the 1 kb upstream of the TSS, except at 200 bp.

We find 740 (~61%) genes in common between the two adapted mutants in Cluster 1 (out of a total of 1223 or 1218 genes in the mutants JMC200-2-5 or JMC160-2-5, respectively). Approximately 13% of these (98) are grouped in the pathway of response to stress according to the Process term of Gene Ontology (GO) Slim Mapper analysis (accessed on 2 October 2024. http://candidagenome.org/cgi-bin/GO/goTermMapper). Additionally, 7.0% are for the response to chemicals, and 2.7% are for cell wall organization (Table S7).

In Clusters 2, 3, and 4, we find that ~50%, 49%, and ~55% of genes, respectively, are common between the two adapted mutants. Approximately 14%, 15%, and 11% of shared genes in Clusters 2, 3, and 4, correspondingly, are grouped in the pathway of response to stress (GO Slim Mapper analysis) (Tables S8–S10). Also, ~8%, 9%, and 8% are for the response to chemical challenge and ~2%, 2%, and 3% are for cell wall organization. Taken together, these data indicate that changes in chromatin accessibility occur at genes with functions that are potentially relevant to drug response.

**Figure 5:**
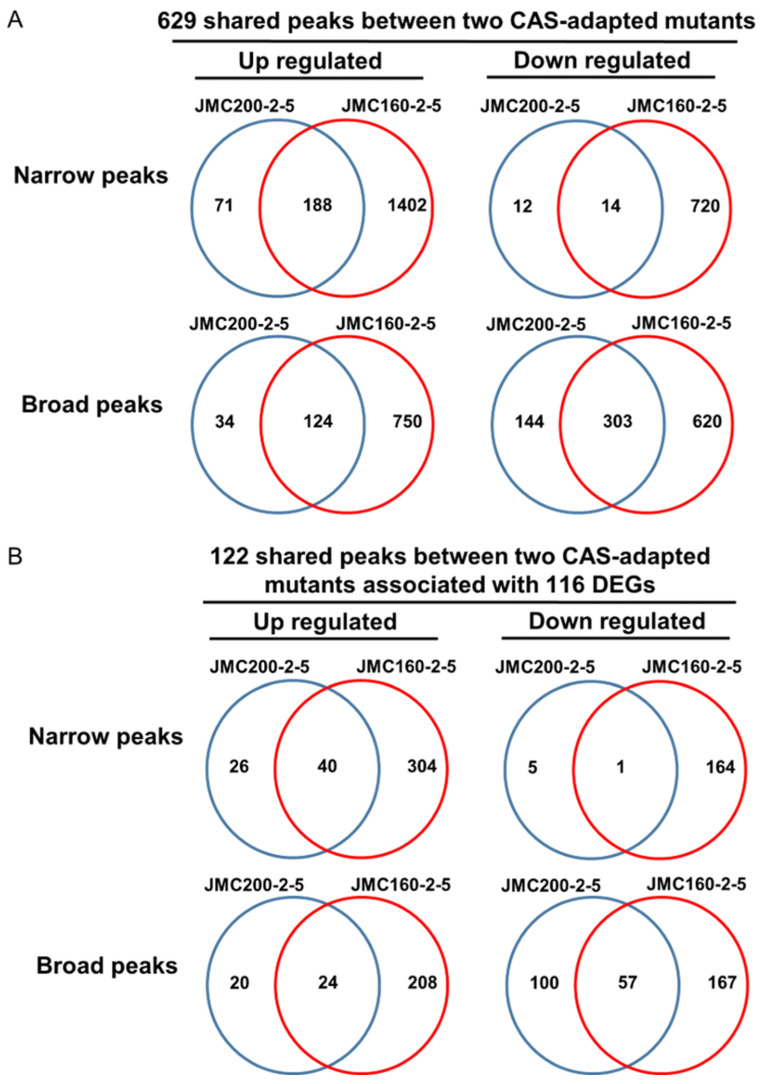
Mutants JMC200-2-5 and JMC160-2-5 share 116 DEGs having 122 ATAC-seq peaks. Shown are Venn diagrams for a total of 629 shared peaks between two mutants (**A**) and 122 shared peaks that correspond to 116 shared DEGs (**B**). In (**B**), intersection areas of diagrams present numbers of shared genes that have peaks. See also Tables S15–S18 for 116 DEGs having 122 ATAC-seq peaks.

DEGs in adapted mutants that exhibit similar changes in accessibility reveal functions important for adaptation. We reasoned that peak-associated differential DEGs shared between two independently derived mutant strains would be more likely to include genes that comprise the basis of adaptation. By using the ChIPseeker Bioconductor package “annotatePeaks” function (Section 4), we find that among the ATAC-seq peaks that differ from the parental strain, 629 are shared between the adapted mutants JMC200-2-5 and JMC160-2-5 (Figure 5A, Tables S11–S14). These include peaks defined both as “broad” and “narrow”, and that differ from the parental strain by gain or loss of accessibility. We find that 122 of these 629 shared differential peaks are found in proximity to 116 DEGs that are similarly changed in the two adapted mutants. Moreover, in each pairing of ATAC-seq peak and DEG, change from the parent strain occurs in the same direction in both adapted mutants, as well as the peaks are predominantly identical in both adapted mutants (Figure 5B, Tables S15–S18).

**Figure 6:**
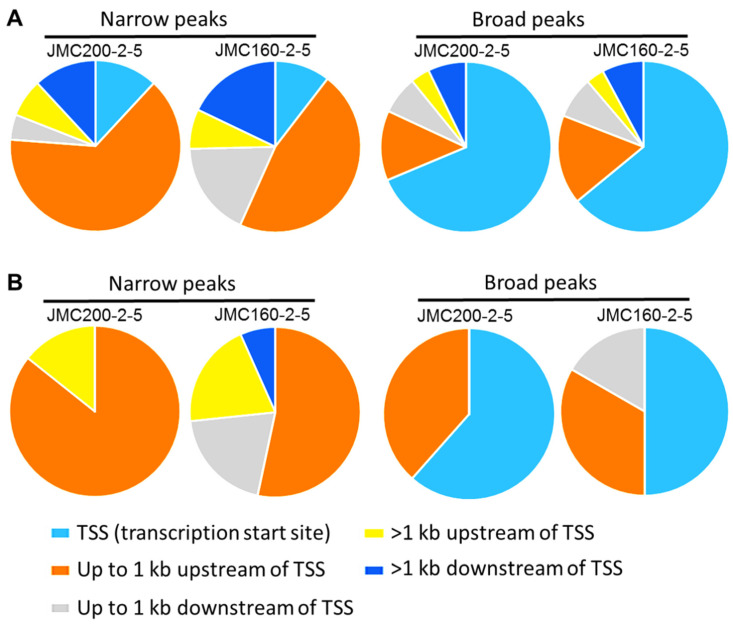
(**A**) Pie charts depict the distribution of 122 ATAC-seq peaks that are found in 116 DEGs common between CAS-adapted mutants JMC200-2-5 and JMC160-2-5. (**B**) Distribution of peaks corresponds to 16 DEGs involved in caspofungin and cell wall remodeling. Note that peaks are found in each DEG, as presented in Tables S15–S18. Some DEGs have multiple peaks. Shown is the distribution of Narrow and Broad peaks over five regions: transcription start site (TSS), up to 1kb upstream and downstream of TSS, and more than 1 kb up or downstream of TSS. Note the substantial difference in distribution between Narrow and Broad peaks. While the majority of Narrow peaks originate at up to 1kb upstream of TSS, the majority of Broad peaks originate at TSS.

We asked how these 116 DEGs that change in the same direction in both mutants are distributed across the K-means clustering of promoters (Figure 4 and Figure 6). As presented in Table 3, we find that 71 of these DEGs appear in the same cluster in each mutant strain: 25 in Cluster 1, 13 in Cluster 2, 14 in Cluster 3, and 19 in Cluster 4. In Cluster 1, all 25 of the shared DEGs are upregulated and 6 of these are involved in cell wall synthesis (*BMT7* and *ATC1*) or sphingolipid transport (*HET1*); or encode fluconazole-downregulated GPI-anchored gene (*PGA25*), MAP kinase (*MKC1*), and scaffold of MAP kinase cascade (*CST5*) (Tables S5 and S6). All 25 shared DEGs could be important for ECN adaptation ([20,29,30,31,32,33,34,35,36]; CGD).

In contrast to Cluster 1, all DEGs in Cluster 4, with or without known function, are downregulated in both mutants. Among DEGs with assigned functions, *UTP15*, *APN2*, or *THR1* are involved with anticandidal drugs from other classes than ECN, while *HRR25* is involved with the response of cell walls to stress [37,38,39].

In Clusters 2 and 3, we find a mixture of upregulated and downregulated DEGs with assigned functions (Table S19). Among these several (*PPH21*, *SEC7*, *NMD5*, *FLU1*; *ECM21*, *CAN2*, *ERG13*) are involved in response to caspofungin or azole drugs, to flucytosine, as well as to amphotericin B (CGD).

Overall, functions were assigned to 74 out of the 116 shared DEGs. We find that these DEGs include some genes potentially important for drug adaptation, including four cell membrane-anchored glycosylphosphatidylinositol (GPI) genes (*IFF6*, *PGA25*, *PGA53*, and *PGA63*) and four genes encoding TFs (*CTF1*, *HAP41*, *CUP9*, and *TRY6*) (Tables S15–S18). In addition, 16 (~14%) of the shared DEGs are implicated in either caspofungin response (9 DEGs) or cell wall synthesis (8 DEGs) according to CGD (Candida Genome Database) (accessed on 15 July 2024, http://www.candidagenome.org/) (Table 4 and Table S20). This suggests that these 116 shared DEGs are likely to be enriched for functions related to adaptation to ECN drugs.

We compiled a list of DEGs associated with caspofungin response and/or cell wall synthesis, and compared the number of differential peaks that occur over or near these genes to the average number of differential peaks associated with genes across the genome (Table 5). We found that genes associated with caspofungin response and/or cell wall synthesis harbor ~3.5-fold more differential peaks per gene, as compared to the rest of the genome.

We determined where these caspofungin response and cell wall synthesis DEGs (16 genes) mapped among the 4 promoter clusters, and did not observe any preferential associations.

Analysis of stress–response pathways in ECN-adapted mutants. We investigated six signaling pathways that are involved in the response to stress by ECN drugs: protein kinase C cell wall integrity (PKC), high osmolality (hog1), cek1, Ca^2+^—calmodulin-activated protein phosphatase calcineurin, molecular chaperone heat shock protein 90, and cAMP-dependent protein kinase A (cAMP-PKA) [40,41,42]. There are 32 genes in the above 6 pathways of which 16 genes are upregulated in each adapted mutant, with 1 gene downregulated in JMC200-2-5 and 4 in JMC160-2-5 (Table S21). There are multiple upregulated genes in all pathways, except the heat shock protein pathway (Table S21). This suggests, as expected, that stress-responsive pathways are upregulated in ECN-adapted mutants.

A total of 16 kinases can be found in 4 stress response pathways listed above (also Table S21). Importantly, a total of 10 out of 16 kinases are upregulated, with expression of the remaining kinases not changing. For example, upregulated and associated with the same peak at TSS, in both adapted mutants, is MAP kinase *MKC1* (Table S21). *MKC1* is responsible for cell wall structure and maintenance, caspofungin response, and cell wall stress ([42,43]; CGD). Another example is *FUN31*, a putative serine/threonine protein kinase, which is involved in cell wall damage response (CGD). Still, another example is *BCY1*, which is a regulatory subunit of protein kinase A, which physically interacts with Tpk1p and is required for nuclear localization of Tpk1p, a catalytic subunit of cAMP-PKA (CGD). *BCY1* and *FUN31* have peaks at TSS and at −259 bp downstream of TSS, respectively, in both mutants (Table S21).

Of several TFs that are known to be activated by stress–response pathways to control cell wall integrity [42], *CRZ1* (orf19.7359) is upregulated in JMC200-2-5, while *RIM101* (orf19.7247) is upregulated in JMC160-2-5 (Tables S5 and S6). Both of these TFs are known to mediate calcium fluctuation in response to alkaline stress [44], and are associated with ATAC-seq peaks (Figure S1F), but the relationship between expression and chromatin accessibility change is not simple.

*CRZ1* encodes a C2H2 zinc finger protein, which is regulated by calcineurin and is involved with drug resistance and cell wall integrity in various fungi [45]. Expression changes of *CRZ1* have been previously demonstrated to be involved in ECN drug tolerance in *C. albicans* [19]. Importantly, *CRZ1* is an auxiliary regulator of the white-opaque phenotypic switch, associated with a change in chromatin accessibility (reviewed in [46]). We previously reported that *CRZ1* is one of only three mutational hot spots found in both adapted mutants, which share three identical *CRZ1* mutations [22].

**Figure 7:**
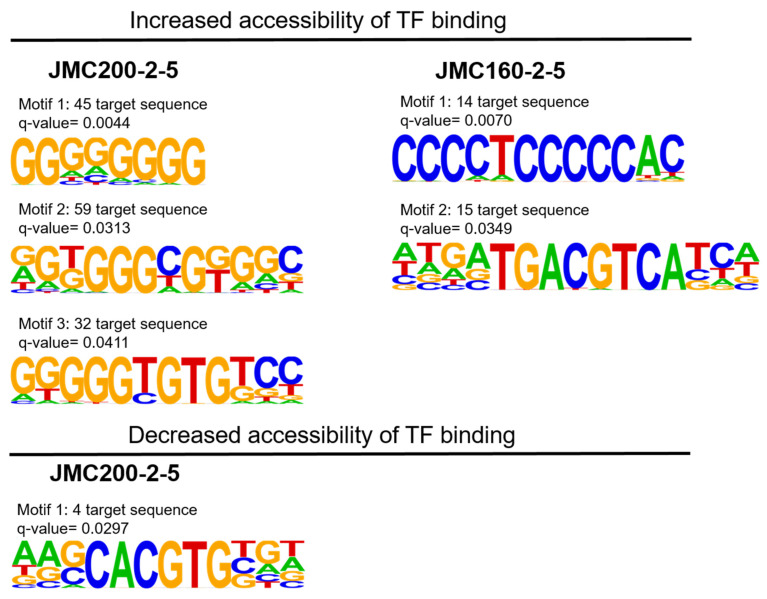
Logos of position weight matrices showing motifs binding sites enriched in differentially accessible TF binding regions from narrow peaks in JMC200-2-5 and JMC160-2-5. The number of matched target sequences as well as the q-value (Benjamini) is indicated in each motif. Top panel increased accessibility and bottom panel decreased accessibility as indicated.

Analysis of TF motifs in regions of differential accessibility. We used HOMER software to search for TF binding motifs in differentially accessible regions in our two mutants (Section 4). For this purpose, we examined only narrow peaks that change significantly in the mutants as compared to the parental strain, since narrow peaks are believed to be associated with TF binding [27]. We used the reference strain SC5314 as a background to determine the significance of candidate motifs. This analysis revealed distinct motifs, three in JMC200-2-5 and two in JMC160-2-5, in regions of increased accessibility (Figure 7, Table S22), along with a single motif enriched in regions of decreased accessibility in JMC200-2-5. The motifs associated with increased accessibility are recognized by zinc finger (Zf) and basic leucine zipper (bZIP) factors.

The binding motif associated with decreased accessibly in JMC200-2-5 corresponds to *S. cerevisiae* Pho4p, a (bHLH) family TF previously shown to be regulated by chromatin accessibility (accessed on 3 August 2024, https://www.yeastgenome.org/locus/S000001930; [47]). In *C. albicans*, Pho4p is involved in stress resistance ([48]; accessed on 3 August 2024, CGD: http://www.candidagenome.org/cgi-bin/locus.pl?locus=pho4&organism=C_albicans_SC5314).

**Figure 8:**
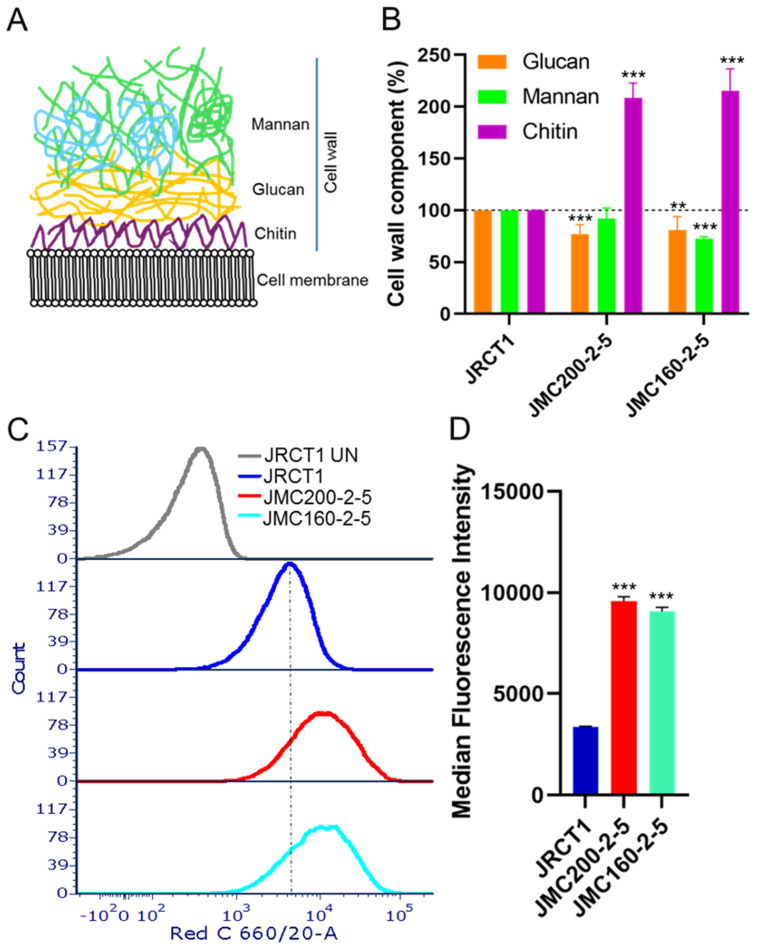
ECN-adapted mutants have remodeled cell walls: (**A**). Cartoon showing three major components of the cell wall: mannan, glucan, and chitin. Mannosylated proteins are represented in blue. (**B**). Changes in mannan, glucan, and chitin amounts of the cell wall in ECN-adapted mutants. Measurements were performed with at least two biological replicates, each replicates with two technical replicates. The amount of glucan, mannan, and chitin in parental strain is set as 100%. The asterisks indicate a *p* value of <0.01 (**), or <0.001 (***), as determined using Student’s *t*-test. (**C**). Increased glucan surface exposure in ECN-adapted mutants was measured with FACS using hDectin-1a and anti-IgG antibody conjugated with Alexa Fluor 647. Shown is a representative histogram of at least a 10^4^ singlet population of caspofungin-adapted mutants in red C channel. JRCT1 UN stands for unstained control. Note that peaks of both mutants JMC200-2-5 and JMC160-2-5 shifted to the right, indicating increased fluorescence as compared with parental JRCT1. (**D**). Bar graph presentation of median fluorescence intensity of each mutant from three biological repeats. The asterisks indicate a *p* value of <0.001 (***), as determined using Student’s *t*-test.

Remodeling of cell walls in ECN-adapted mutants. The *C. albicans* cell wall consists of three major polysaccharides: chitin, glucan, and mannan (Figure 8A). We asked if chromatin accessibility is correlated with the expression of genes involved in biosynthesis and remodeling of the cell wall. These include glucan, chitin, and mannan synthesis genes, as well as regulatory genes on Ch2 and Ch5 (Table S23) [14,15,35,49,50,51,52,53,54,55,56]. We curated a list of 52 genes known to be involved in cell wall biosynthesis. Among these, we find that 39 and 48 are DEGs in JMC200-2-5 and JMC160-2-5, respectively. A total of 35 (67%) are shared.

In JMC200-2-5 and JMC160-2-5, among the 52 cell-wall-associated genes, 11 and 28, respectively, were identified as DEGs that are associated with ATAC-seq peaks, which differ significantly in intensity compared to the parent strain (Table S23). Eight of these (*CHS1*, *CHS3*, *PMT1*, *PMT2*, *MNS1*, *BMT1*, *BMT7*, and *HAS1*) are common to both mutants. We note that for *BMT7*, *MNS1*, and *HAS1*, there is a similar correlation between accessibility and expression change in both mutants.

*BMT1*, which is downregulated in both mutants, and *BMT7*, which is upregulated in both mutants, each correlate with two associated ATAC-seq peaks; *HAS1*, which is downregulated on Ch5 in both mutants, correlates with one broad peak. A more complex association with peaks is observed in *CHS3*: the direction of change in *CHS3* expression correlates with changes in a broad peak in JMC160-2-5, but not in JMC200-2-5. Interestingly, Chs3p synthesizes the majority of chitin in yeast (>50%) and hyphal (>60%) *C. albicans* [57]. We do not have a simple explanation for the complexity of this enzyme regulation, which will require more study in the future.

As ECN-adapted mutants harbor multiple DEGs that are associated with the cell wall (Table S23), we asked whether adaptation to ECNs is coupled with remodeling of the cell wall. We measured the amount of chitin, glucan, and mannan in JMC200-2-5 and JMC160-2-5 vs. their parental strain (Section 4) and found significant changes. Specifically, glucan decreased and chitin increased in both mutants, while mannan decreased in JMC160-2-5 (Figure 8B).

We also determined the cell surface exposure of the pathogenic epitope glucan and found that its exposure significantly increased (Figure 8C,D). Previous studies showed that *CHT2*, *RPS25B*, *UAP1*, *URA7*, *HAS1*, *CKS1*, and orf19.4149.1, which are DEGs in both mutants, act to increase glucan exposure, i.e., unmasking glucan [15]. Two out of seven of these DEGs are associated with ATAC-seq peaks: *HAS1* has a broad peak at TSS in both mutants, while *CHT2* has a broad peak at TSS in JMC160-2-5. Of note, transcriptional and epigenetic changes in *HAS1* correlate.

Overall, we find significant cell wall remodeling in ECN-adapted mutants, and that DEGs controlling the increase in glucan cell surface exposure are associated with changes in chromatin accessibility.

## 3. Discussion

In *C. albicans*, chromatin accessibility has previously been implicated in yeast-to-hyphae transition, biofilm formation, response to oxidative stress, nitrogen assimilation, and virulence [28,58,59,60]. We previously reported the underlying mutagenesis and multiple genome-wide expression changes that accompany adaptation to ECN drugs [14,22]. In this study, we asked if chromatin accessibility similarly contributes to the adaptation of *C. albicans* to ECN drugs in two independently derived euploid mutants by employing ATAC-seq. We find that drug adaptation is coupled with quantitative genome-wide changes in chromatin accessibility, as reflected in differential increases or decreases in peak intensities in the adapted mutants, as compared to the parental strain. Our results are similar to those of a previous study [28], which found that adaptation to oxidative stress in *C. albicans* resulted in genome-wide alterations in chromatin accessibility. Moreover, we find extensive similarities between two mutant strains, reflected in similar changes in similarly positioned peaks of chromatin accessibility, as well as in gene expression patterns, which suggests that adaptation is associated with common changes in gene regulation and chromatin remodeling.

We also find strong correlations between changes in chromatin accessibility in ECN-adapted mutants and differential gene expression as determined by RNA-seq. We find that regions of differential accessibility occur predominantly at promoter regions, a substantial number of which are associated with DEGs. As also reported in other studies, however [28,61,62], not all DEGs are associated with changes in promoter-proximal chromatin accessibility, which could reflect regulatory function from a distance [63], changes in post-transcriptional regulation, or other stages of regulation independent of chromatin structure as measured by ATAC-seq.

More evidence for the contribution of chromatin accessibility to ECN drug adaptation comes from the association of ATAC-seq peaks with genes that are involved with drug adaptation and are shared between both adapted mutants. In 16 peak-associated DEGs that are involved in either caspofungin response or cell wall synthesis, each DEG contains the associated peak at the identical position in both adapted mutants, despite independent origins of the mutant strains. Similarly, the positions of shared peaks and correlation of changes between ATAC-seq peak and DEG are also found among DEGs from other groups of genes, including *MKC1*, *FUN31*, and *BCY1* that are involved in stress–response pathways or *BMT1*, *BMT*7, and *HAS1* that are responsible for cell wall biosynthesis and remodeling.

ECN drugs target the cell wall of *C. albicans*. Both of the adapted mutants that we analyzed exhibit similarly remodeled cell walls, including increased chitin—potentially via cell wall salvage [40]—and decreased glucan with increased cell surface exposure—potentially controlled via simultaneous adaptive changes in the expression of 15 genes residing on Ch2 (all genes upregulated) or Ch5 (all genes downregulated) [14,15]. While there are similarities in changed gene expression between the mutant strains that correlate with this, there are also differences, which suggest that while the resulting phenotypic changes that underlie drug adaptation are common between strains, the precise alterations in gene expression that achieve those changes can vary in complicated ways. Previously, we obtained evidence that adaptation to ECNs may result, at least in part, from altered expression of genes that control cell wall remodeling. We reported independent knockouts of 15 genes residing on either chromosome 2 or chromosome 5 from the genome of the parental strain control cell wall remodeling, as well as the minimum inhibitory concentration of ECN [14,15]. Taken together, our data imply that remodeling of the cell wall, an important organelle targeted by ECN drugs, contributes to ECN adaptation.

Our ATAC-seq datasets reveal substantial differences in chromatin accessibility in ECN-adapted mutant strains of *C. albicans*, which are entirely quantitative in nature, involving changes in peak intensity as opposed to peak location or occurrence. Combined with similar quantitative changes that emerge from the RNA-seq datasets, this suggests that drug adaptation in the mutants that we analyzed entails broad-based but not absolute changes in gene expression, correlated with underlying changes in the efficiency of chromatin remodeling at gene promoters. Insofar as such changes ultimately originate with DNA mutations in the adapted strains, it seems likely that mutations in transcription factors or cofactors that alter but do not eliminate TF function should underlie the adapted phenotype. Among the mutations that we previously characterized in these adapted strains, those that occur in known or predicted TFs in both strains, such as *CRZ1*, and in particular, those that further correlate with DNA binding motifs associated with differentially accessible regions will therefore be of particular interest in future studies. In general, our application of the ATAC-seq assay to ECN-adapted *C. albicans* mutants suggests that differences in chromatin accessibility contribute to adaptation.

## 4. Materials and Methods

Strains, medium, and growth conditions. *C. albicans* ECNs-adapted mutants, JMC200-2-5 and JMC160-2-5 used in the study, were generated by exposing a parental strain JRCT1 to caspofungin added to solid medium in independent parallel experiments using two different concentrations of caspofungin (200 ng/mL and 160 ng/mL), as reflected in mutants’ names [21]. The genome wide DNA changes by DNA-seq and transcription profile by RNA-seq were published for these mutants [14,22], but transcriptional profiles were reanalyzed here (see below). Cells were stored, maintained, and grown using a standardized approach to prevent induction of chromosome instability [64,65]. Briefly, cells were stored in a 25% (vol/vol) glycerol solution at −80 °C to interrupt metabolism and routinely grown at 37 °C.

YPD medium contained 1% yeast extract, 2% peptone, and 2% dextrose [66].

RNA-seq analysis. RNA-seq fastq files were downloaded from GSE217094. Quality filtering and adapter removal are performed using FastP version 0.23.1 with the following parameters: “--length_required 35 --cut_front_window_size 1 --cut_front_mean_quality 13 --cut_front --cut_tail_window_size 1 --cut_tail_mean_quality 13 --cut_tail -y –r” [67]. Processed and cleaned reads were then mapped to the *C. albicans* SC5314 Assembly 22 using STAR_2.7.9a with the following parameters: “—twopass Mode Basic --runMode alignReads --outSAMtype BAM Unsorted—outSAMstrandField intronMotif --outFilterIntronMotifs RemoveNoncanonical –outReadsUnmapped Fastx” [68,69]. Gene level read quantification was derived using the subread-2.0.1 package (featureCounts) with a GTF annotation file (SC5314 Assembly 22) and the following parameters for stranded RNA libraries “-s 2 -t exon -g gene_id” [70]. Differential expression analysis was performed using DESeq2-1.34.0 with a *p*-value threshold of 0.05 within R version 3.5.1 (https://www.R-project.org/) [71].

Nuclei preparation for ATAC-seq. *C. albicans* grown in YPD were used for nuclei preparation based on published protocol with minor modifications [28]. The cells of the mutants JMC200-2-5 and JMC160-2-5 and of parental JRCT1 were streaked on YPD agar plates and incubated at 37 °C till young colonies of approximate size 1 × 105 to 3 × 105 cells per colony grew up. The colonies were picked and suspended in sterile water, and cells were counted with the help of a hemacytometer. A total of 2 × 106 cells were inoculated in 10 mL of YPD medium and incubated at 37 °C, 220 rpm for 4.5 h in an incubator-shaker. The cells were harvested, washed with 1X sorbitol buffer [1.4 M sorbitol, 40 mM HEPES-KOH pH 7.5, 0.5 mM MgCl_2_, 10 mM DTT (dithiothreitol)], and counted with aid of a hemacytometer. A total of 3 × 10^7^ cells were pooled and suspended in 330 µL of sorbitol buffer of which 192 µL was transferred to a fresh microcentrifuge tube, 8 µL of 50 mg/mL of 100T zymolyase (U.S. Biological, Swampscott, MA, USA) was added, and the microcentrifuge tube was incubated at 30 °C for 10 min on mixer.

Spheroplasting efficiency was observed by measuring the OD_600_ of 10 µL of zymolyase-treated cells suspended in 1 mL of water. More than a 90% drop in OD_600_ as compared to the control suggests good spheroplasting efficiency. The spheroplasts were harvested at 5000 g at 4 °C for 10 min and washed with 1× ice-cold sorbitol buffer (without DTT). The pellet was gently suspended in 500 µL of 1× sorbitol buffer (without DTT) and counted. A total of 5 × 10^6^ spheroplasts were transferred to a fresh microcentrifuge tube, centrifuged at 5000 g at 4 °C for 10 min, and subjected to tagmentation.

Genomic DNA (gDNA) isolation from parental JRCT1. The parental strain JRCT1 was inoculated in a 50 mL Falcon tube containing 10 mL of YPD medium and grown at 37 °C for 16 h with shaking at 220 rpm. The cells from 1.5 mL cultures were harvested and washed twice with water following centrifugation at 5000 rpm for 3 min. The cells were resuspended in 500 µL of 1× buffer (0.9 M sorbitol, 0.1 M EDTA pH 7.5) in which 10 µL of 10 mg/mL zymolyase was added and incubated at 37 °C for 90 min. The suspension was centrifuged at 13,000 rpm for 2 min and the pellet was resuspended in 350 µL 1× Tris-EDTA buffer (50 mM Tris-HCl pH 7.5, 20 mM EDTA) and 40 µL of 10% SDS. The cell lysate was vortexed and incubated at 65 °C for 30 min, 100 µL of 5M KOAc was added, and the sample was incubated at 4 °C for 10 min. The sample was centrifuged at 13,000 rpm for 10 min at room temperature and the supernatant was transferred to a fresh microcentrifuge tube. The supernatant was mixed with two volumes of 100% ethanol and centrifuged at 13,000 rpm for 10 min at 4 °C. The gDNA pellet was treated with 5 µL of RNase A (10 mg/mL) (Sigma-Aldrich, St. Louis, MO, USA) at 37 °C for 30 min, precipitated again with 100 µL of 5M KOAc and 1 mL of 100% ethanol. The gDNA pellet was resuspended in 200 µL nuclease-free water and concentration was measured with a nanodrop spectrophotometer (Thermo Fisher Scientific, Waltham, MA, USA) as per manufacturer’s instructions.

Tagmentation. Illumina tagment DNA TDE1 enzyme and buffer kit (Illumina, San Diego, CA, USA) was used for tagmentation. A total of 50 µL of the tagmentation reaction was prepared by adding 25 µL TD buffer (Tagment DNA buffer) (2×), 4 µL of TDE1 (Tagment DNA enzyme 1), 0.5 µL of 1% digitonin, 1 µL of protease inhibitor (50×), and 19.5 µL of nuclease-free water. A total of 5 million spheroplasts of each JMC200-2-5 and JMC160-2-5, and parental JRCT1, was re-suspended in 50 µL of the tagmentation reaction; in addition, 300 ng (1 µL) of control naked gDNA was re-suspended in 49 µL of the tagmentation reaction. The samples were mixed gently by pipetting and were incubated at 37 °C for 30 min with agitation on the mixer. To stop the reaction, 300 µL of ERC buffer (Elute Reaction Cleanup buffer) (Qiagen, Hilden, Germany) was added. Samples were stored at −20 °C before transferring to the University of Rochester Genomic Research Center (URGRC) where the tagmentation reaction was purified using a MinElute reaction cleanup kit (Qiagen, Hilden, Germany).

ATAC-seq library amplification, size selection, and next-generation sequencing. Three independent cell cultures of parental JRCT1 and ECNs-adapted mutants JMC200-2-5 and JMC160-2-5 were grown and used to prepare ATAC-seq libraries as three biological replicates. Tagmented DNA was isolated with the Qiagen MinElute Reaction Cleanup kit (Qiagen, Hilden, Germany) and eluted in 20 μL. An aliquot of 10 μL was used to perform an initial 5 cycles of library PCR amplification using Illumina RNA/DNA Unique Dual index primers, with the following cycling conditions: 98 °C for 45 s enzyme activation; 98 °C for 15 s, 63 °C for 30 s, 72 °C for 1 min for 5 cycles; and a final hold step at 4 °C. A 5 μL aliquot of the PCR reaction was used to perform quantitative polymerase chain reaction (qPCR) on a QuantStudio 12K Flex (Thermo Fisher, Waltham, MA, USA) to determine how many additional cycles of PCR amplification should be performed on each sample [72]. Following qPCR, PCR amplification of the libraries continued for the calculated number of additional cycles using the cycling parameters above. ATAC-seq libraries in this study were amplified within a range of 8–17 total PCR cycles. The amplified ATAC-seq libraries were immediately purified and size selected with a double-sided solid-phase reversible immobilization (SPRI) approach (0.5X/1.4X) using AMPure XP beads (Beckman Coulter, Indianapolis, IN, USA). Library quantity and quality were assessed by Qubit and Bioanalyzer (Agilent, Santa Clara, CA, USA) assays, respectively, with paired-end 50 nt sequencing performed on NovaSeq 6000 (Illumina, San Diego, CA, USA) at URGRC [72].

ATAC-seq data analysis workflow.

a. Pre-processing and read alignment. Raw reads generated from the Illumina basecalls were demultiplexed using bcl2fastq version 2.19.1. Quality filtering and adapter removal were performed using FastP version 0.23.2 with the following parameters “--trim_poly_g -x --cut_window_size 4 -3 -l 35” [67]. Processed/cleaned reads were then mapped to the Assembly 22 of the *C. albicans* reference strain SC5314 (accessed on 7 December 2023, https://www.ncbi.nlm.nih.gov/datasets/taxonomy/237561) using bowtie2 2.4.4 (-x ${REF} -X 2000 -p ${CPUS} -1 ${FASTQ1} -2 ${FASTQ2} -S ${SAM}) [73]. Insert sizes were determined using Picard (2.17.0) CollectInsertSizeMetrics (Picard Toolkit, Broad Institute, 2019). Read fragments less than 100 bp were filtered for using Deeptools (3.1.3) alignmentSieve “--ignoreDuplicates --minMappingQuality 10 --minFragmentLength 0 --maxFragmentLength 100” and were considered to be nucleosome-free regions for potential binding of TSs [74]. Replicate bam files were merged with samtools (1.9) merge and bigWigs were created using Deeptools (3.1.3) bamCoverage “--binSize 1 --normalizeUsing CPM” [75].

b. Genomic coverage of ATAC-seq signals, and heatmap clustering. Deeptools “bamCompare” was used to compute the log2Ratio of mutant compared to control. The bigwigs were then used in Deeptools “computeMatrix” (“--referencePoint TSS -b 1000 -a 200”) to compute scores for each gene. The output matrix was subsequently used to generate a profile plot using Deeptools “plotProfile” (“--kmeans 4”) and a cluster heatmap using Deeptools “plotHeatmap” (“--zMin -1 --zMax 1 --kmeans 4”).

c. Peak calling. Narrow peak enrichments were called using the MACS2 algorithm (version 2.2.7.1) with the following ATAC-seq specific parameters “--nomodel --shift -100 --extsize 200” and a *p*-value threshold of 0.05 for individual samples. To determine broad peaks, we used the “--broad” parameter of MACS2, which combines nearby narrow peaks and uses a *p*-value threshold of 0.1 to allow for the detection of a more diffuse signal [76].

d. Analysis of differential ATAC-seq peaks and genomic annotation of ATAC-seq peaks. Differential chromatin accessibility between mutants and controls was determined using DiffBind and DESeq2 packages [71,77]. Regions with a *p*-adjusted value < 0.05 were considered to be significant. PCA (principal component analysis) plots (Figure S2) and heatmaps were created within the DiffBind package. Volcano plots were generated using GraphPad Prism 9.5.0. A GTF file for *C. albicans* SC5314 Assembly 22 was downloaded from NCBI and a TxDb object was created using the “makeTxDbFromGFF” function in GenomicFeatures [78]. Peaks were annotated using ChIPseeker “annotatePeaks” function (tssRegion = c(-1000, 200), genomicAnnotationPriority = c(’Promoter’, ’5UTR’, ’3UTR’, ’Exon’, ’Intron’, ’Downstream’, ’Intergenic’) [79]. Pie charts for annotated regions were created in ChIPseeker.

e. Motif Analysis. Motif analysis was conducted with HOMER findMotifs using the differential accessible bed files from DiffBind (“${BED} ${FASTA} ${OUTPUT} -size given -mask) (accessed on 17 July 2024, http://homer.ucsd.edu/homer/motif/). HOMER software is designed to analyze sequence motifs of various organisms including mammals, as well as *S. cerevisiae*. Known motifs were considered significant with a *p*-adjusted value < 0.05.

Determination of glucan content in the cell wall. Cell wall glucan was determined by an aniline blue assay [14]. Briefly, cells were grown at 37 °C for 19 h on YPD plates from −80 °C stock for independent colonies. Cells were collected from YPD plates and counted with a hemocytometer. A total of 10^6^ cells from each strain were further processed for glucan solubilization. Cell wall glucan was solubilized by 1M NaOH at 80 °C for 30 min. Aniline blue mixture [0.03% aniline blue, 0.18 M HCl, 0.49 M glycine-NaOH (pH 9.5)] was added to the solubilized glucan and incubated for 50 °C for 30 min and then 30 min at room temperature. Fluorescence was measured in a plate reader (Spectra Max; Molecular Devices Corp., Sunnyvale, CA, USA) at an excitation wavelength of 400 nm and an emission wavelength of 469 nm, with a cutoff of 455 nm.

Determination of chitin content in the cell wall. Cell wall chitin was determined by measuring glucosamine released by acid hydrolysis of a dry cell wall, as described previously [14]. Briefly, cells were grown from −80 °C stock on YPD plates at 37 °C for 19 h. Cells were harvested, washed with water, and suspended in sorbitol lysis buffer [1 M sorbitol, 0.1 M EDTA (pH 7.4)]. Cells were disrupted by vortexing with 0.5 mm glass beads. The cell lysate (pellet) was washed 5 times with 1M NaCl. Remaining cell wall proteins were removed with SDS-mercaptoethanol extraction buffer (50 mM Tris, 2% SDS, 0.3 M mercaptoethanol) by incubation at 100 °C for 10 min. The cell wall pellet was washed with distilled water, air-dried, and weighed. Dry cell wall was incubated with 1 mL of 6M HCl at 100 °C for 17 h. The acid hydrolysates were dried by incubating at 65 °C for 25 h. The hydrolysate was dissolved in 1 mL of distilled water. Then, 100 μL of each sample was mixed with 100 μL of 1.5 M Na_2_CO_3_ in 4% acetyl acetone and incubated at 100 °C for 20 min. In the next step, 700 μL of 96% ethanol and 100 μL of a p-dimethylaminobenzaldehyde solution in a 1:1 mixture of ethyl alcohol and concentrated HCl were added. The mixture was incubated at room temperature for 1 h. The absorbance at 520 nm was measured in a plate reader (Spectra Max; Molecular Devices Corp., Sunnyvale, CA, USA). Glucosamine (Sigma-Aldrich, St. Louis, MO, USA) was used to prepare the standard curve for chitin measurement. The chitin level was calculated as a percentage of the cell wall dry weight for each sample.

Determination of mannan content in the cell wall. Cell wall mannan measurements were performed with the alcian blue staining method [14]. Briefly, cells were grown from a −80 °C stock on YPD plates at 37 °C for 19 h. Cells were collected and counted with a hemocytometer. Approximately 1.4 × 10^7^ cells were collected and washed with distilled water. Next, cells were suspended in 1 mL of 30 μg/mL of alcian blue (Sigma-Aldrich, St. Louis, MO, USA) in 0.02 M HCl (pH 3.0) and incubated at room temperature for 10 min with orbital shaking. After incubation, cell suspension was centrifuged and the supernatant was collected. The absorbance at 620 nm was measured for 100 μL of the supernatants collected from each sample using a plate reader (Spectra Max; Molecular Devices Corp., Sunnyvale, CA, USA). A standard curve was plotted to determine the concentration of alcian blue. Alcian blue binding per cell [picogram (pg) per cell] was calculated according to the formula x = [(u − v)/*n*] × 10^6^, where x is the bound alcian blue (pg/cell), u is the original alcian blue concentration used for staining (µg/mL), v is the alcian blue concentration in the supernatant (µg/mL), and *n* is the number of cells used for staining.

Determination of glucan surface exposure by Fluorescence-Activated Cell Sorting (FACS). Glucan exposure was performed as described previously [15]. Cells were grown from a −80 °C stock on YPD plates at 37 °C for 19 h. Briefly, cells were collected and counted with a hemocytometer. Approximately 3.5 × 10^6^ cells were collected and washed twice with 1× PBS (phosphate-buffered saline). Next, primary staining of cells was performed with 0.5 µg/mL of hDectin-1a (catalog no. fc-hdec1a) (InvivoGen, San Diego, CA, USA) at 25 °C for 1 h with shaking. Cells were washed twice with 1× PBS. Secondary staining of cells was performed by incubating 4 mg/mL of goat-raised Anti-human IgG antibody (catalog no. A-21445) (Invitrogen, Waltham, MA, USA) conjugated with Alexa Fluor 647 at 25 °C for 30 min in the dark with shaking. Cells were washed thrice with 1× PBS to remove any unbound antibodies. After washing, cells were suspended in 1 mL of 1× PBS for FACS analysis. FACS data collected from LSRII/Fortessa/Symphony A1 (Becton, Dickinson and Company, NJ, USA) were analyzed by software FCS Express 7. FACS data were recorded from three biorepeats, and each repeat contained 30,000 events. The singlet population of at least 20,000 cells was selected for further analysis of glucan exposure.

## Figures and Tables

**Table 1:** Distribution of significant ATAC-seq peaks over chromosomes in ECN-adapted mutants JMC200-2-5 and JMC160-2-5.

Ch	Peaks in JMC200-2-5				Peaks in JMC160-2-5			
	Broad		Narrow		Broad		Narrow	
	Gain	Loss	Gain	Loss	Gain	Loss	Gain	Loss
1	34	115	48	10	179	233	300	191
2	22	47	32	5	140	133	243	98
3	15	59	39	4	106	129	205	102
4	6	47	26	2	89	97	173	78
5	12	53	26	0	71	79	144	66
6	19	35	22	1	68	63	131	67
7	21	29	26	0	61	57	125	37
R	29	62	40	4	160	132	269	95
Total in column	158	447	259	26	874	923	1590	734
Total	605		285		1797		2324	

**Table 2:** Percent of ATAC-seq peaks that are associated with DEGs and changing in the same direction as DEGs in ECN-adapted mutants JMC200-2-5 and JMC160-2-5.

Mutant	Total DEGs	Total ATAC-Seq Peaks	% Peaks and Associated DEGs Changing in the Same Direction
JMC200-2-5	2664	890	24.26
JMC160-2-5	3135	4121	23.41

**Table 3:** Distribution of 71 shared and peaks-associated DEGs over Clusters 1–4 in caspofungin-adapted mutants JMC200-2-5 and JMC160-2-5.

Cluster	JMC200-2-5	JMC160-2-5	DEGs in Common	
			Total	With Function/No Function
1	33	27	25	11/14
2	22	35	13	9/4
3	32	21	14	10/4
4	22	33	19	13/6

**Table 4:** List of 9 caspofungin-responsive genes and 8 genes involved in cell wall synthesis that carry ATAC-seq peaks and are shared DEGs in ECN-adapted mutants JMC200-2-5 and JMC160-2-5. Shown are expression changes by RNA-seq and changes in associated ATAC-seq peaks, as determined by ratio mutant/parent.

Standard Name	Assembly 19/21 Identifier	Systematic Name	RNA-Seq Ratio		ATAC-Seq Peak Ratio			
			JMC200-2-5	JMC160-2-5	JMC200-2-5		JMC160-2-5	
					Narrow	Broad	Narrow	Broad
Caspofungin responsive genes								
NA *	orf19.6578	C7_01510W_A	2.52	3.12	1.21	NA	1.27	NA
*ECM21*	orf19.4887	C1_10180C_A	2.03	1.63	1.21	1.1	1.25	NA
*LSP1*	orf19.3149	C2_06730W_A	1.28	1.25	1.18	NA	1.17	NA
*PPH21*	orf19.1683	C3_01600W_A	1.66	2.06	1.22	NA	1.19	NA
*MKC1* **	orf19.7523	CR_00120C_A	1.46	1.72	NA	1.08	1.18	1.11
*HAS1*	orf19.3962	C5_04750C_A	0.56	0.35	NA	0.9	NA	0.87
*CAN2* ^†^	orf19.111	C6_01060C_A	0.36	0.36	NA	0.94	0.75 0.83	0.88
*PHO87*	orf19.2454	C1_05940W_A	0.76	0.49	NA	0.89	NA	0.73
*ERG13*	orf19.7312	CR_09160C_A	0.39	0.37	NA	0.94	NA	0.94
Cell wall synthesis genes								
*GLC3*	orf19.5622	C6_03340C_A	1.93	2.20	1.19	1.1	1.24	NA
*BMT7*	orf19.342	C3_03450C_A	2.18	1.91	1.2	1.18	1.35	1.32
*FUN31*	orf19.7451	C3_06620W_A	1.89	1.54	1.29	NA	1.25	NA
*MKC1* **	orf19.7523	CR_00120C_A	1.46	1.72	NA	1.08	1.18	1.11
*ATC1*	orf19.6214	C1_06940C_A	1.57	1.92	NA	1.09	1.19	1.19
*PDE2* ^‡^	orf19.2972	C1_02840W_A	0.64	0.72	NA	0.92	NA	0.8 1.17
*HRR25*	orf19.3476	C6_02340W_A	0.78	0.67	NA	0.92	0.81	0.81
*BMT1* ^†^	orf19.6782	C3_07180C_A	0.40	0.38	NA	0.92	0.77 0.83	0.8

* NA stands for Not Applicable. ** *MKC1* belongs to both categories of genes according to CGD. ^†^ Stands for two narrow peaks in JMC160-2-5. ^‡^ Stands for two broad peaks in JMC160-2-5.

**Table 5:** Ratio differential peaks/genes in 16 caspofungin-responsive and cell wall synthesis genes vs. the entire genome in caspofungin-adapted mutants JMC200-2-5 and JMC160-2-5.

Mutant	Number of Peaks	Number of Genes	Ratio (Peaks/Genes)
Caspofungin-responsive and cell wall synthesis genes			
JMC200-2-5	19	16	1.2
JMC160-2-5	25	16	1.6
Entire genome in *C. albicans* caspofungin-adapted mutants			
JMC200-2-5	890	6182	0.1
JMC160-2-5	4121	6182	0.7

## Data Availability

The original data presented in the study are openly available in the Gene Expression Omnibus under accession number GSE284528.

## Supplementary Materials

The following supporting information can be downloaded at: https://www.mdpi.com/article/10.3390/jof11020110/s1. Figure S1. Snapshots showing examples of loss and gain of broad and narrow peaks as indicated. Shown are IGV tracks for mutants JMC160-2-5 and JMC200-2-5, parental JRCT1, and control gDNA, as indicated. (**A**–**G**) show examples of various genes having peaks: (**A**) *PGA25*, *PGA63*; (**B**) *LSP1*, *ECM21*; (**C**) *PHO87*, *CAN2I*; (**D**) *GLC3*, *BMT7*; (**E**) *MKC1*, *FUN31*; (**F**) *CRZ1*, *RIM101*; (**G**) *CUP9*, *CHS1*. Figure S2. Principle component analysis. Shown are parental JRCT1 and caspofungin-adapted mutants JMC200-2-5 and JMC160-2-5. Narrow and Broad peaks are indicated. Table S1. Analysis of ATAC-seq narrow peak of JMC200-2-5. Table S2. Analysis of ATAC-seq broad peak of JMC200-2-5. Table S3. Analysis of ATAC-seq narrow peaks of JMC160-2-5. Table S4. Analysis of ATAC-seq broad peaks of JMC160-2-5. Table S5. DEseq2 analysis of JMC200-2-5. Table S6. DEseq2 analysis of JMC160-2-5. Table S7. GO slim mapper of shared genes in Cluster 1 of both adapted mutants. Table S8. GO slim mapper of shared genes in Cluster 2 of both adapted mutants. Table S9. GO slim mapper of shared genes in Cluster 3 of both adapted mutants. Table S10. GO slim mapper of shared genes in Cluster 4 of both adapted mutants. Table S11. A total of 188 shared upregulated narrow peaks between JMC200-2-5 and JMC160-2-5. Table S12. A total of 14 shared downregulated narrow peaks between JMC200-2-5 and JMC160-2-5. Table S13. A total of 124 shared upregulated broad peaks between JMC200-2-5 and JMC160-2-5. Table S14. A total of 303 shared downregulated broad peaks between JMC200-2-5 and JMC160-2-5. Table S15. Correspondence of 40 shared upregulated narrow peaks and DEGs between JMC200-2-5 and JMC160-2-5. Table S16. Correspondence of 1 shared downregulated narrow peak and DEG between JMC200-2-5 and JMC160-2-5. Table S17. Correspondence of 24 shared upregulated broad peaks and DEGs between JMC200-2-5 and JMC160-2-5. Table S18. Correspondence of 57 shared downregulated broad peaks and DEGs between JMC200-2-5 and JMC160-2-5. Table S19. Distribution of 71 DEGs over Clusters 1–4 in caspofungin-adapted mutants JMC200-2-5 and JMC160-2-5. Table S20. List of genes involved in caspofungin response and cell wall remodeling. Table S21. RNA-seq and ATAC-seq analysis of genes involved in stress-responsive pathways. Table S22. Summary table of significant motifs analyzed from Homer software. Table S23. A list of DEGs that are involved with synthesis or control of cell wall components.
