# Supplementary material for: Echinocandin Adaptation in Candida albicans Is Accompanied by Altered Chromatin Accessibility at Gene Promoters and by Cell Wall Remodeling
Source: J Fungi (Basel). 2025 Feb 1;11(2):110. doi: 10.3390/jof11020110 (PMC11856910; doi:10.3390/jof11020110)
Supplement: Supplementary file 1 [file jof-11-00110-s001.zip › List of supplementary tables.pdf]

List of supplementary tables

Table S1. Analysis of ATAC-seq narrow peak of JMC200-2-5
Table S2. Analysis of ATAC-seq broad peak of JMC200-2-5
Table S3. Analysis of ATAC-seq narrow peaks of JMC160-2-5
Table S4. Analysis of ATAC-seq broad peaks of JMC160-2-5
Table S5. DEseq2 analysis of JMC200-2-5
Table S6. DEseq2 analysis of JMC160-2-5
Table S7. GO slim mapper of shared genes in Cluster 1 of both adapted mutants
Table S8. GO slim mapper of shared genes in Cluster 2 of both adapted mutants
Table S9. GO slim mapper of shared genes in Cluster 3 of both adapted mutants
Table S10. GO slim mapper of shared genes in Cluster 4 of both adapted mutants
Table S11. 188 shared upregulated narrow peaks between JMC200-2-5 and JMC160-2-5
Table S12. 14 shared downregulated narrow peaks between JMC200-2-5 and JMC160-2-5
Table S13. 124 shared upregulated broad peaks between JMC200-2-5 and JMC160-2-5
Table S14. 303 shared downregulated broad peaks between JMC200-2-5 and JMC160-2-5
Table S15. Correspondence of 40 shared upregulated narrow peaks and DEGs between JMC200-2-5 and JMC160-2-5
Table S16. Correspondence of 1 shared downregulated narrow peak and DEG between JMC200-2-5 and JMC160-2-5
Table S17. Correspondence of 24 shared upregulated broad peaks and DEGs between JMC200-2-5 and JMC160-2-5
Table S18. Correspondence of 57 shared downregulated broad peaks and DEGs between JMC200-2-5 and JMC160-2-5
Table S19. Distribution of 71 DEGs over Clusters 1-4 in caspofungin-adapted mutants JMC200-2-5 and JMC160-2-5
Table S20. List of genes involved in caspofungin response and cell wall remodeling
Table S21. RNA-seq and ATAC-seq analysis of genes involved in stress responsive pathways
Table S22. Summary table of significant motifs analyzed from Homer software.
Table S23. A list of DEGs that are involved with synthesis or control of cell wall components.
