# Supplementary material for: Echinocandin Adaptation in Candida albicans Is Accompanied by Altered Chromatin Accessibility at Gene Promoters and by Cell Wall Remodeling
Source: J Fungi (Basel). 2025 Feb 1;11(2):110. doi: 10.3390/jof11020110 (PMC11856910; doi:10.3390/jof11020110)
Supplement: Supplementary file 1 [file jof-11-00110-s001.zip › Table S22.pdf]

Table S22. Summary table of significant motifs analyzed from Homer software.

Name*	q-value by Benjamini method	Number of target sequences with Motif	% of target sequences with motif	Number of background sequences with motif	% of background sequences with motif
JMC200-2-5, increased chromatin accessibility due to gain of Narrow peak<br>Total target sequences=259, total background sequences=29604					
Maz (Zf)	0.0044	45	17.37	2534.1	8.56
Klf14 (Zf)	0.0313	59	22.78	4082.4	13.79
Klf10 (Zf)	0.0411	32	12.36	1801.9	6.09
JMC160-2-5, increased chromatin accessibility due to gain of Narrow peak<br>Total target sequences=1591, total background sequences=30200					
Zfp281 (Zf)	0.007	14	0.88	60.4	0.2
Tga3 (bZIP)	0.0349	15	0.94	84.7	0.29
JMC200-2-5, decreased chromatin accessibility due to loss of Narrow peak<br>Total target sequences=26, total background sequences=40522					
Pho4 (bHLH)	0.0297	4	15.38	297.6	0.69
JMC160-2-5, decreased chromatin accessibility due to loss of Narrow peak<br>Total target sequences=728, total background sequences=44211					
NS	NS	NS	NS	NS	NS

* Name of a protein with motif according to HOMER database.

NS stands for No Significant motifs.

Zf stands for zinc finger.

bZIP stands for basic leucine zipper.

bHLH stands for basic helix-loop-helix.
