# Supplementary material for: Echinocandin Adaptation in Candida albicans Is Accompanied by Altered Chromatin Accessibility at Gene Promoters and by Cell Wall Remodeling
Source: J Fungi (Basel). 2025 Feb 1;11(2):110. doi: 10.3390/jof11020110 (PMC11856910; doi:10.3390/jof11020110)
Supplement: Supplementary file 1 [file jof-11-00110-s001.zip › Table S23.pdf]

Table S23. A list of DEGs that are involved with synthesis or control of cell wall components. Presented are expression changes by RNA-seq calculated as mutant to parent ratio. Note that all shared genes have expression change in same direction in two adapted mutants except for three genes.

Standard Name	Assembly 19/21 Identifier	Ch	RNS-seq				Common DEG	ATAC-seq			
			JMC200-2-5		JMC160-2-5			JMC200-2-5		JMC160-2-5	
			Ratio mutant /parent	FDR	Ratio mutant /parent	FDR		Narrow	Broad	Narrow	Broad
**Glucan synthesis genes**											
FKS1	orf19.2929	1	NS	NS	0.7	0.03		NS	NS	1.2	0.9
FKS3	orf19.2465	1	1.8	0.00	1.5	0.03	*FKS3*	NS	NS	NS	NS
**Chitin synthesis genes**											
CHS1	orf19.5188	7	0.6	0.01	0.2	0.00	*CHS1*†	1.2	1.1	2.1	1.3
CHS2	orf19.7298	R	2.8	0.00	1.5	0.00	*CHS2*	NS	0.9	NS	NS
*CHS3**	orf19.4937	1	1.3	0.00	0.8	0.00	*CHS3*†	NS	0.9	NS	0.9
CHS5	orf19.807	2	1.6	0.00	1.6	0.00	*CHS5*	NS	NS	NS	1.1
CHS7	orf19.2444	1	1.4	0.01	NS	NS		NS	NS	NS	NS
CHS8	orf19.5384	3	NS	NS	0.4	0.00		NS	NS	NS	NS
CHT4	orf19.1515	2	NS	NS	1.6	0.02		NS	NS	NS	1.1
**Mannan synthesis genes**											
PMT1	orf19.5171	7	1.4	0.01	1.7	0.00	*PMT1*†	NS	0.9	NS	0.77
PMT2	orf19.6812	3	1.7	0.00	2.2	0.00	*PMT2*†	NS	0.9	NS	0.88
PMT4	orf19.4109	2	NS	NS	1.7	0.00		NS	NS	1.2	NS
PMT6	orf19.3802	4	0.7	0.02	0.6	0.00	*PMT6*	NS	NS	NS	NS
MNT1	orf19.1665	3	NS	NS	1.6	0.00		NS	NS	NS	0.88

CWH41	orf19.4719	4	NS	NS	1.5	0.00		NS	NS	NS	NS
MNS1	orf19.1036	1	1.6	0.00	2.4	0.00	*MNS1*†	NS	0.9	NS	0.95
MNN1	orf19.4279	5	NS	NS	0.7	0.00		NS	NS	NS	NS
MNN2	orf19.2347	1	0.6	0.00	0.6	0.00	*MNN2*	NS	NS	1.2	1.12
MNN4	orf19.2881	4	0.7	0.01	NS	NS		0.9	NS	0.8	0.86
MNN12	orf19.4900	1	2.3	0.00	2.6	0.00	*MNN12*	NS	NS	NS	NS
MNN14	orf19.6996	3	0.3	0.00	0.5	0.00	*MNN14*	NS	NS	NS	NS
MNN15	orf19.753	1	NS	NS	0.6	0.00		NS	NS	NS	NS
MNN22	orf19.3803	4	0.6	0.00	0.3	0.00	*MNN22*	NS	0.96	NS	NS
MNN24	orf19.1995	2	3.5	0.00	3.3	0.00	*MNN24*	NS	NS	0.8	NS
MNN26	orf19.6692	7	0.7	0.02	NS	NS		NS	NS	NS	0.91
BMT1	orf19.6782	3	0.4	0.00	0.4	0.00	*BMT1*†	NS	0.92	0.8	0.8
RHD1	orf19.54	1	NS	NS	0.6	0.00		NS	NS	NS	NS
BMT3	orf19.3282	R	2.4	0.00	1.7	0.00	*BMT3*	NS	NS	0.8	NS
BMT4	orf19.5612	6	NS	NS	0.6	0.00		NS	0.92	0.8	0.77
BMT5	orf19.1464	2	1.8	0.00	NS	NS		NS	NS	NS	NS
*BMT6**	orf19.5602	6	1.4	0.01	0.6	0.00	*BMT6*	NS	NS	NS	NS
BMT7	orf19.342	3	2.2	0.00	1.9	0.00	*BMT7*†	1.2	1.2	1.3	1.32
MNT3	orf19.1010	R	0.6	0.01	0.5	0.00	*MNT3*	NS	NS	1.2	NS
ALG1	orf19.4410	4	1.5	0.04	1.8	0.00	*ALG1*	NS	NS	NS	NS
ALG6	orf19.1843	R	NS	NS	2.1	0.00		NS	NS	NS	0.87
ALG7	orf19.2187	2	NS	NS	0.7	0.04		NS	NS	NS	1.09
ALG11	orf19.3468	6	0.4	0.00	0.6	0.01	*ALG11*	NS	NS	NS	NS
*CHK1**	orf19.896	2	1.4	0.01	0.7	0.01	*CHK1*	NS	NS	NS	NS
**Regulatory genes on Ch2 and Ch5**											
CHT2	orf19.3895	5	0.3	0.00	0.2	0.00	*CHT2*	NS	NS	NS	1.1
URA7	orf19.3941	5	0.4	0.00	0.2	0.00	*URA7*	NS	NS	NS	NS
RPO26	orf19.2643	5	0.4	0.00	0.2	0.00	*RPO26*	NS	NS	NS	0.9
HAS1	orf19.396	5	0.6	0.00	0.3	0.00	*HAS1*†	NS	0.9	NS	0.9
DUS4	orf19.966	5	0.7	0.01	0.4	0.00	*DUS4*	NS	NS	1.2	NS

RPS25B	orf19.6663	5	0.6	0.00	0.5	0.00	*RPS25B*	NS	NS	NS	NS
UAP1	orf19.4265	5	0.6	0.00	0.5	0.00	*UAP1*	NS	NS	NS	NS
CKS1	orf19.1282	5	0.5	0.00	0.6	0.00	*CKS1*	NS	NS	NS	NS
NS	orf19.4149.1	5	0.5	0.00	0.5	0.00	Orf19.4149.1	NS	NS	NS	NS
NS	orf19.970	5	NS	NS	0.5	0.00		NS	NS	NS	NS
ECS1	orf19.1766	2	2.1	0.03	2.1	0.03	*ECS1*	NS	NS	NS	NS
ECS3	orf19.5833	2	1.3	0.01	1.6	0.00	*ECS3*	NS	NS	0.8	NS
PR26	orf19.5793	2	1.4	0.00	1.9	0.00	*PR26*	NS	NS	NS	1.1
LEU42	orf19.1375	2	1.5	0.01	1.6	0.00	*LEU42*	NS	NS	1.1	NS

* Genes that changed their expression in opposite direction in two adapted mutants.

† Common DEGs having peaks in both adapted mutants.

NS stands for Not Significant.
