# Supplementary figures and images for: Echinocandin Adaptation in Candida albicans Is Accompanied by Altered Chromatin Accessibility at Gene Promoters and by Cell Wall Remodeling
Source: J Fungi (Basel). 2025 Feb 1;11(2):110. doi: 10.3390/jof11020110 (PMC11856910; doi:10.3390/jof11020110)

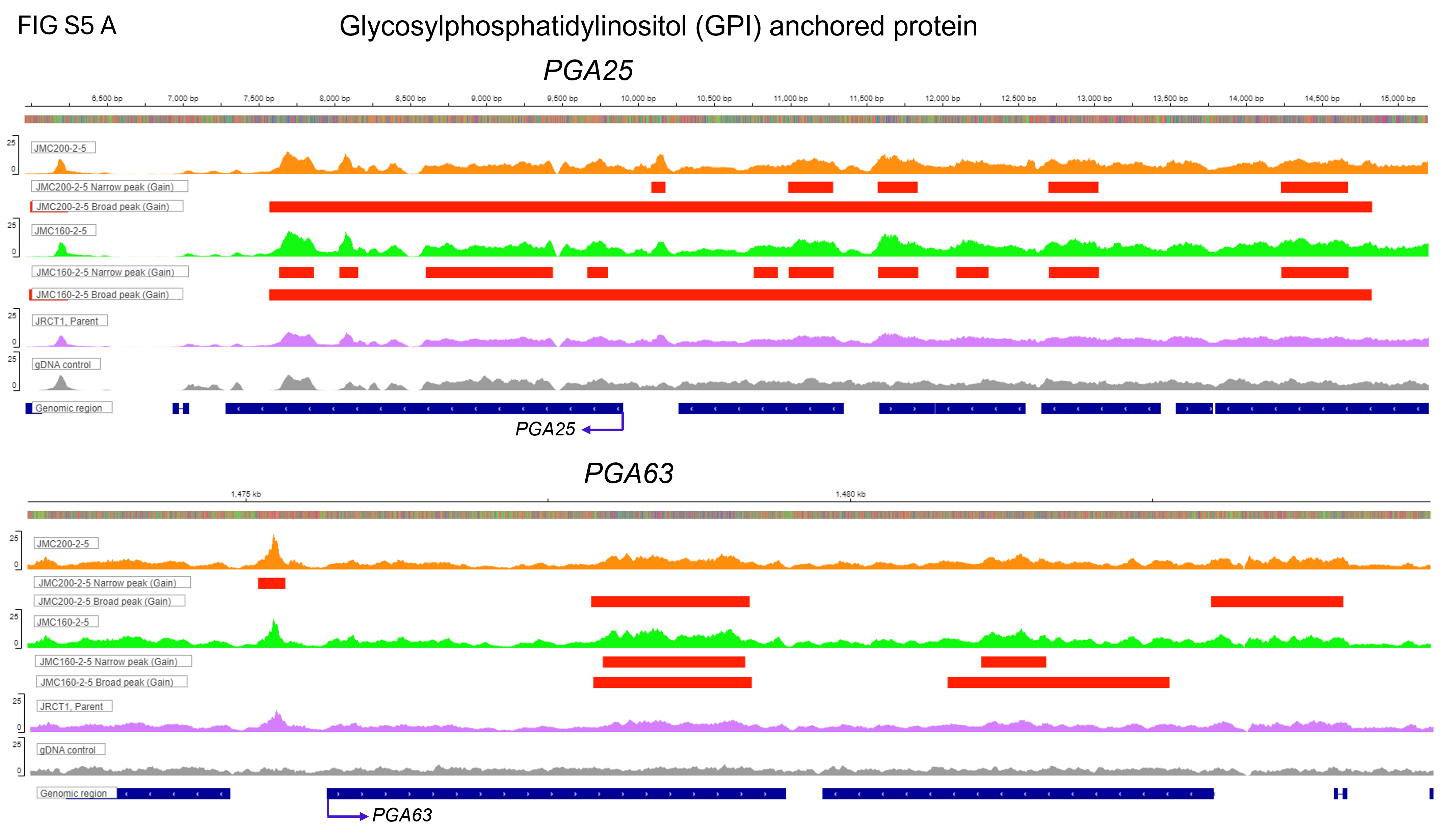

Supplement: Supplementary file 1 [file jof-11-00110-s001.zip › FIG S1 A (GPI acnhored genes).tif]

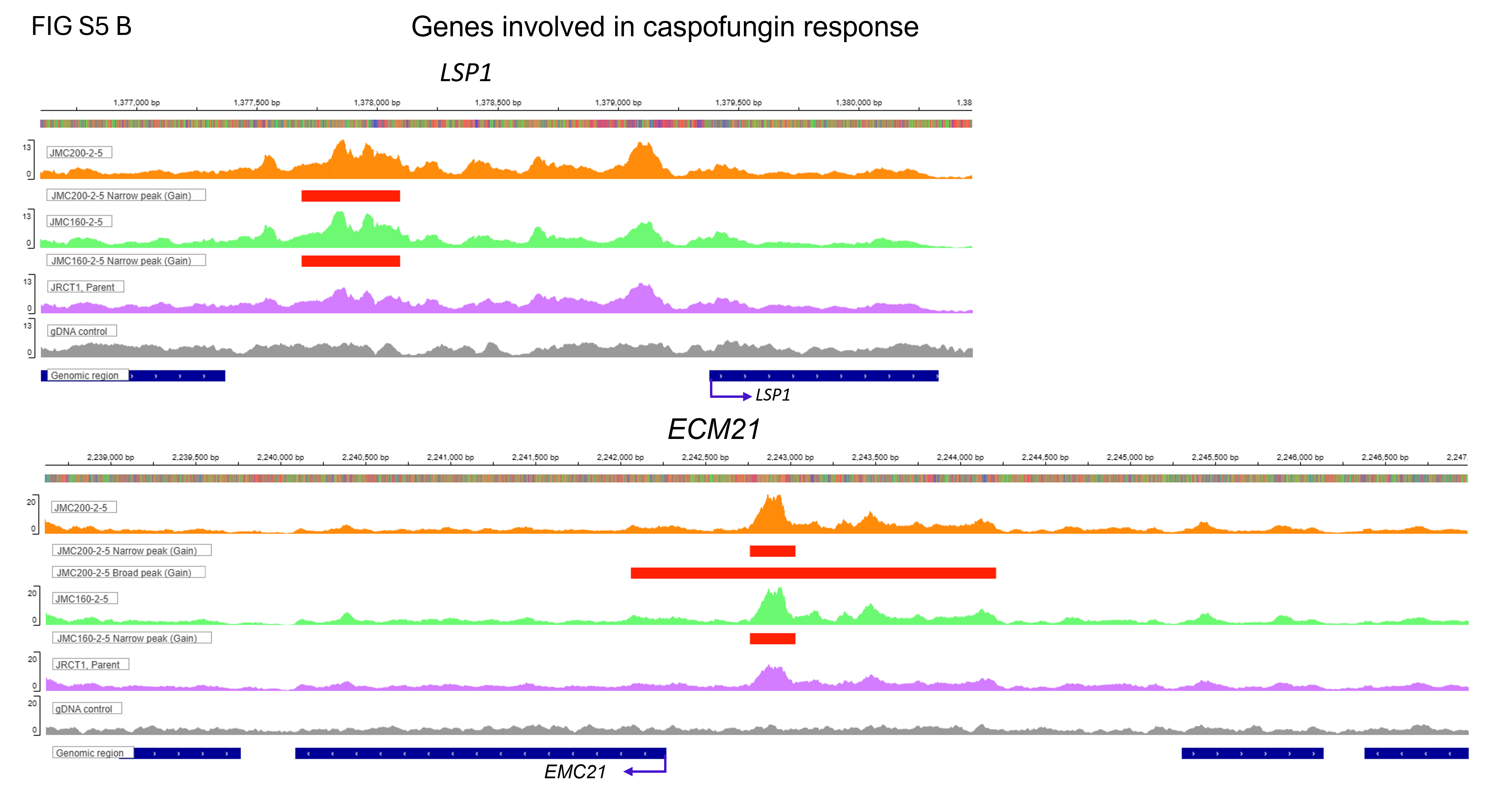

Supplement: Supplementary file 1 [file jof-11-00110-s001.zip › FIG S1 B (Genes involved in caspofungin response).tif]

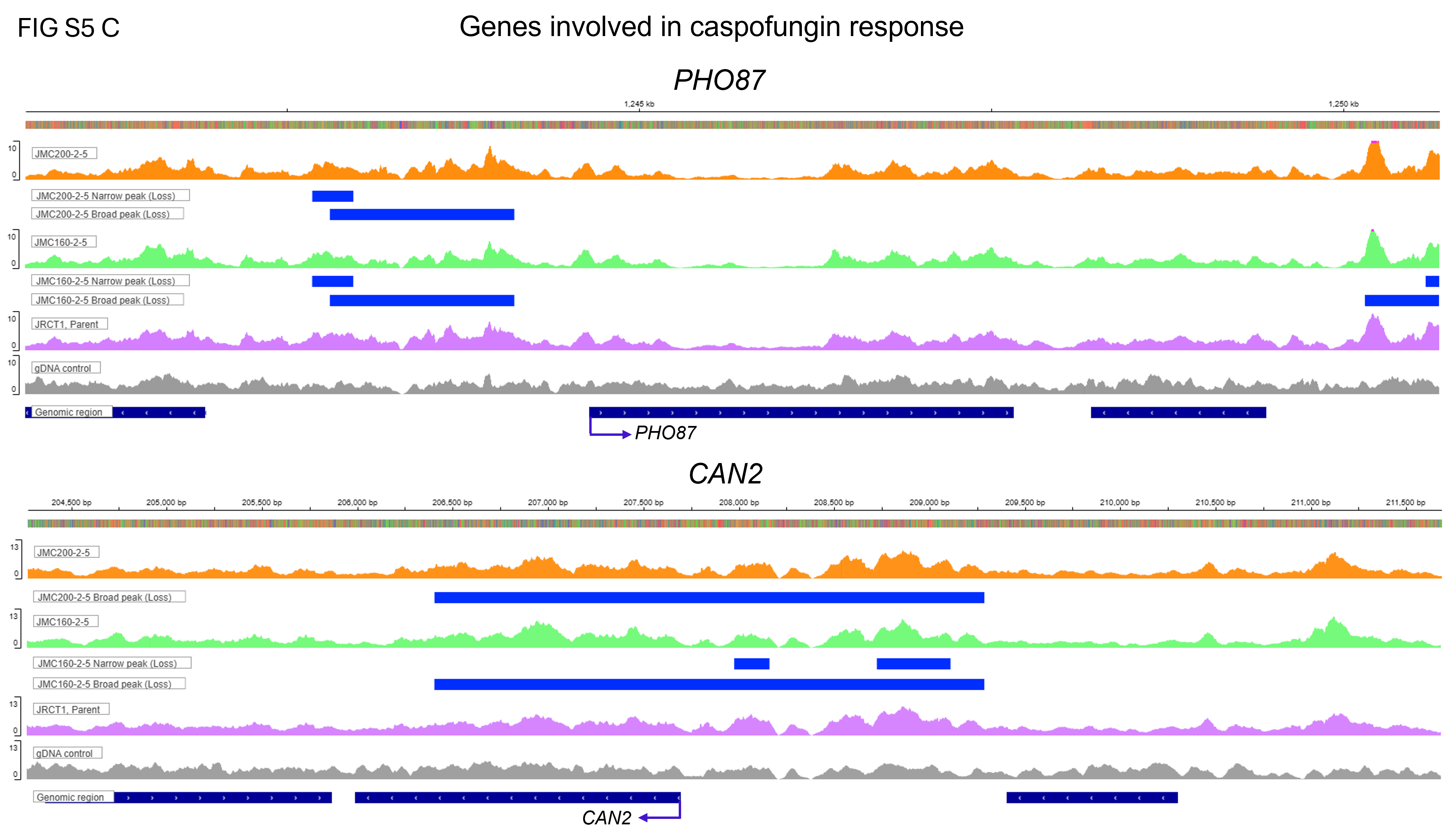

Supplement: Supplementary file 1 [file jof-11-00110-s001.zip › FIG S1 C (genes involved in caspofungin response).tif]

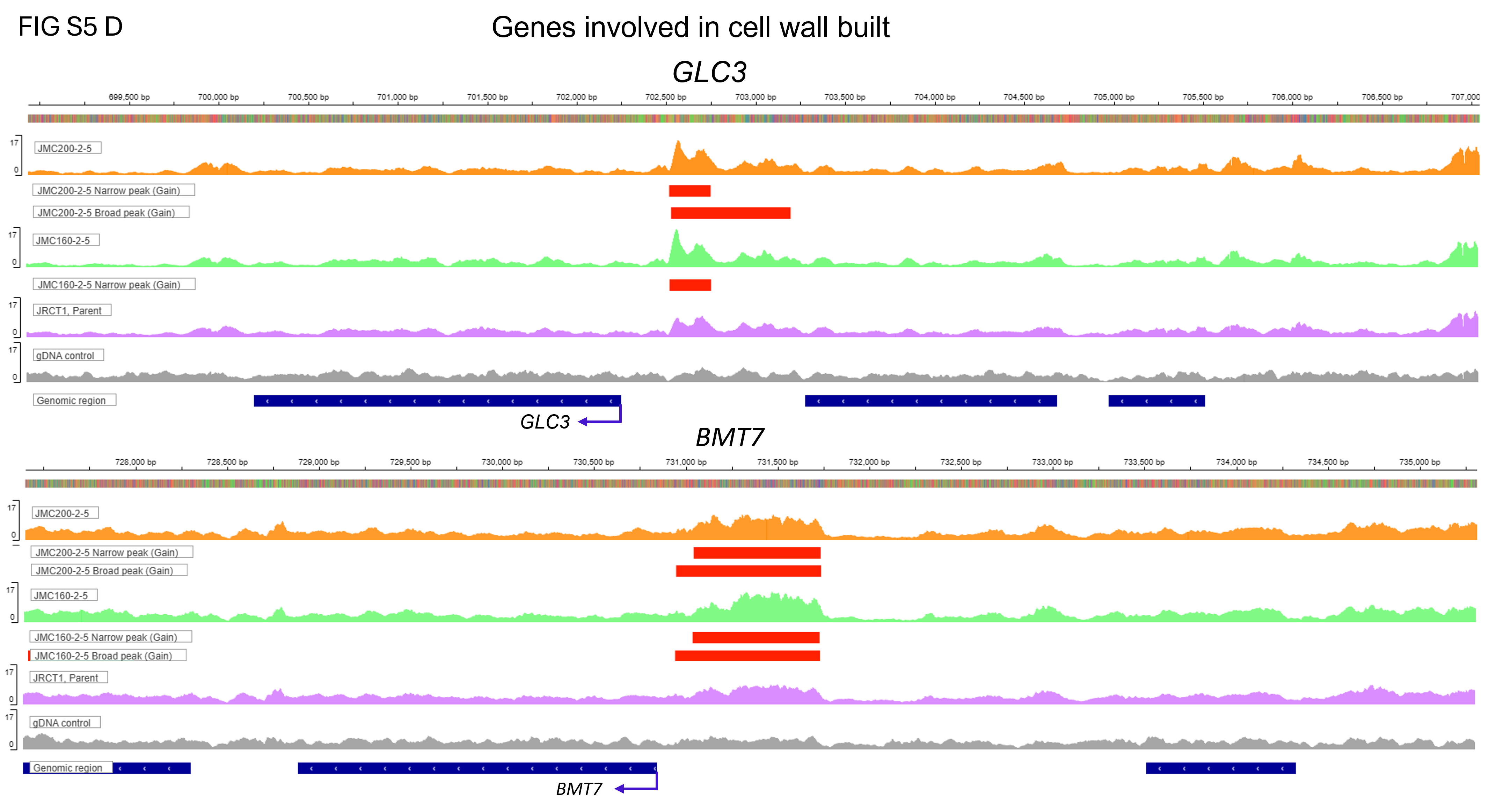

Supplement: Supplementary file 1 [file jof-11-00110-s001.zip › FIG S1 D (Genes involved in cell wall build).tif]

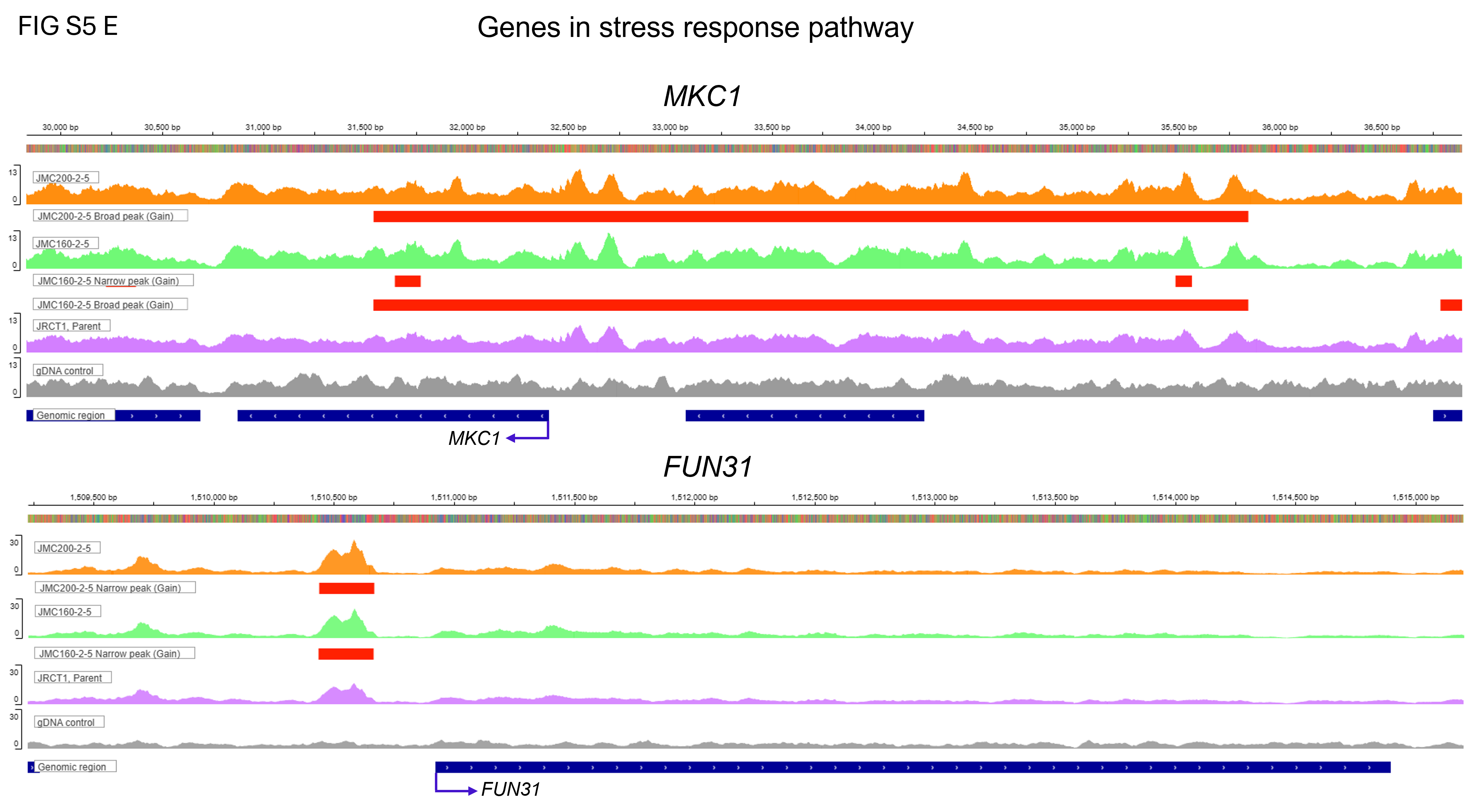

Supplement: Supplementary file 1 [file jof-11-00110-s001.zip › FIG S1 E (Genes in stress response pathway).tif]

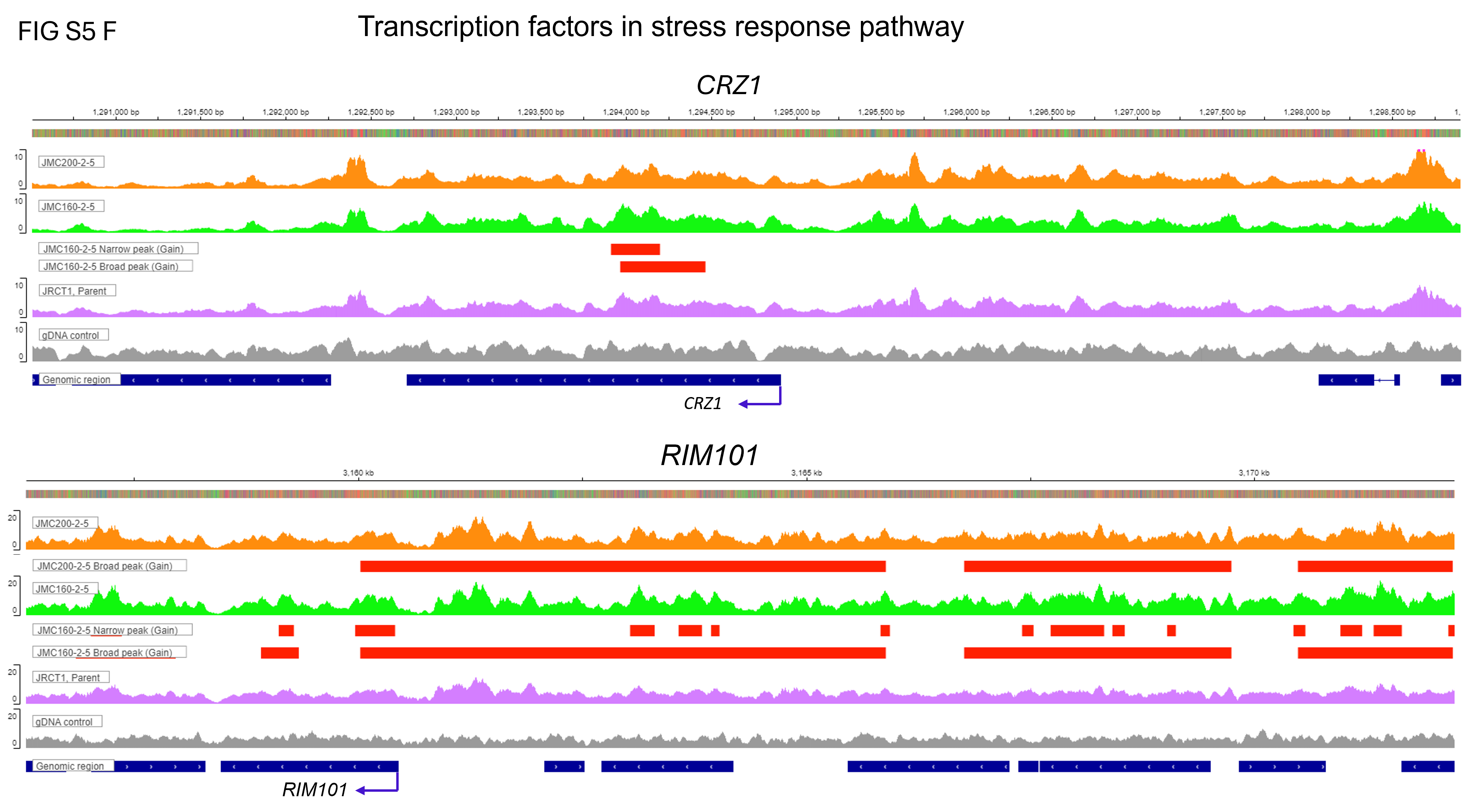

Supplement: Supplementary file 1 [file jof-11-00110-s001.zip › FIG S1 F (Transcription factors in stress response pathway).tif]

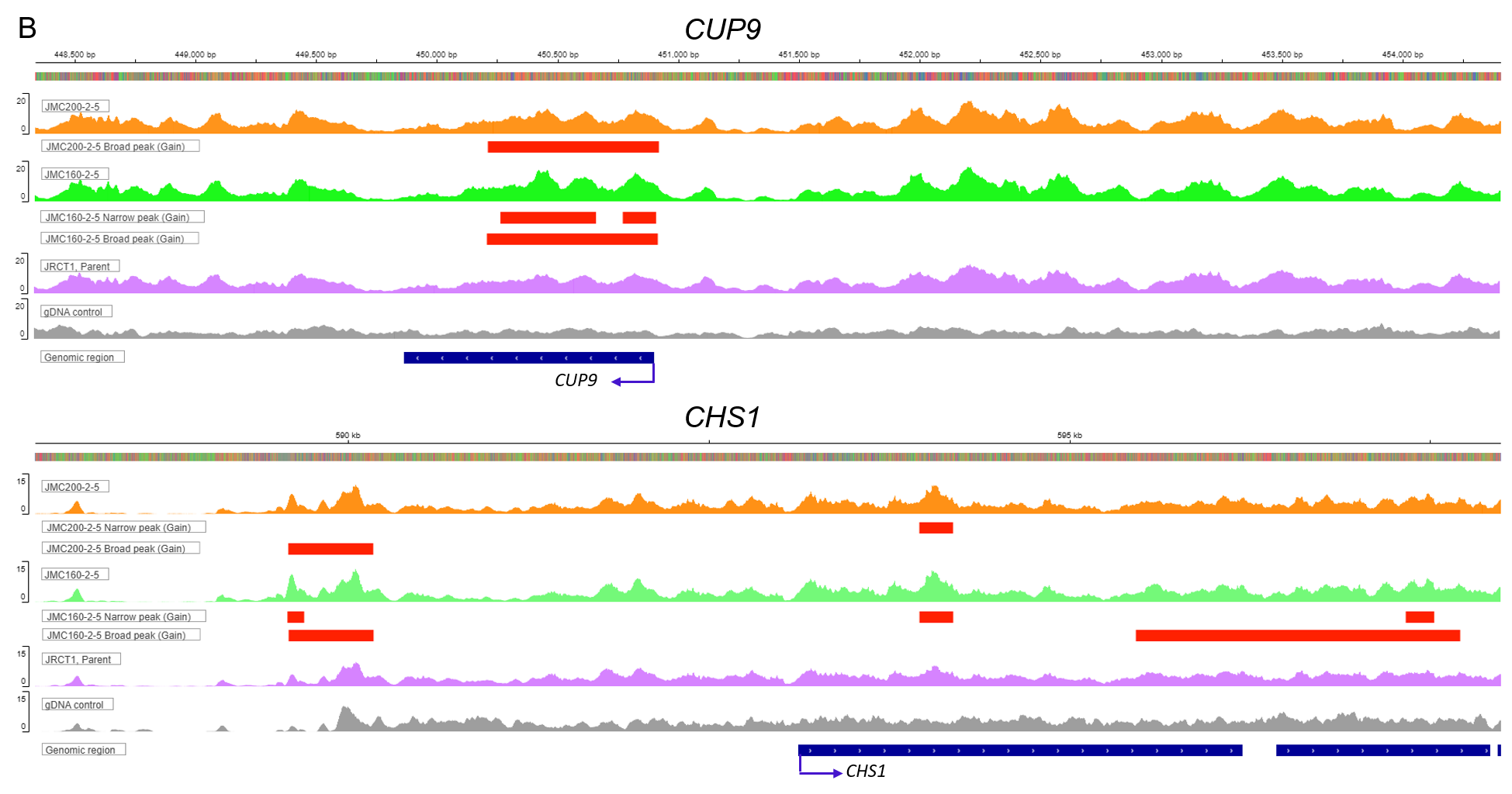

Supplement: Supplementary file 1 [file jof-11-00110-s001.zip › FIG S1 G (CUP9 CHS1).tif]

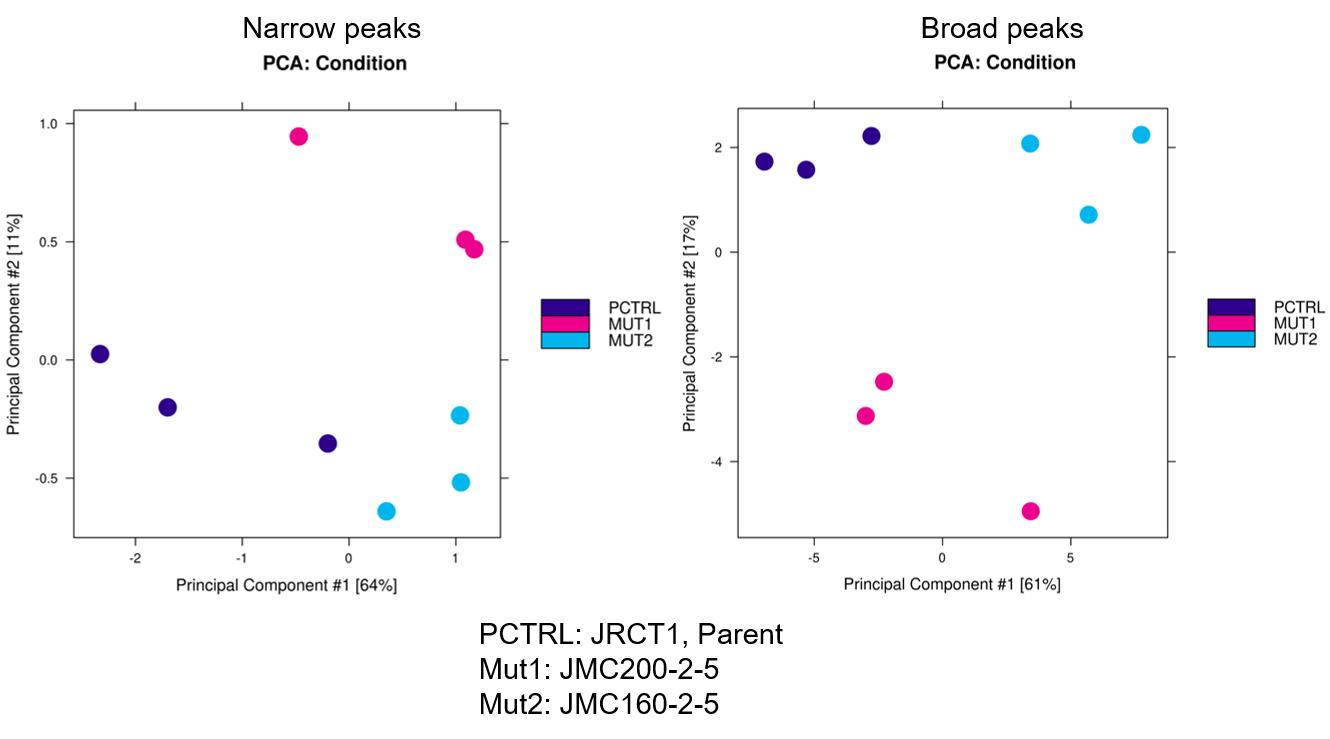

Supplement: Supplementary file 1 [file jof-11-00110-s001.zip › FIG S2 (principle component analysis).tif]
